# Mice prenatally exposed to valproic acid do not show autism-related disorders when fed with polyunsaturated fatty acid-enriched diets

**DOI:** 10.1038/s41598-023-38423-z

**Published:** 2023-07-11

**Authors:** Valentine Turpin, Maud Schaffhauser, Mathieu Thabault, Agnès Aubert, Corinne Joffre, Eric Balado, Jean-Emmanuel Longueville, Maureen Francheteau, Christophe Burucoa, Maxime Pichon, Sophie Layé, Mohamed Jaber

**Affiliations:** 1 Université de Poitiers, Inserm, Laboratoire de Neurosciences Expérimentales et Cliniques, Poitiers, France; 2 Université de Poitiers, Inserm, PHAR2, Poitiers, France; 3 CHU de Poitiers, Poitiers, France; 4 Université de Bordeaux, INRAE, Bordeaux INP, NutriNeurO, UMR 1286, Bordeaux, France

**Keywords:** Autism spectrum disorders, Risk factors

## Abstract

Dietary supplementations with n-3 polyunsaturated fatty acid (PUFA) have been explored in autism spectrum disorder (ASD) but their efficiency and potential in ameliorating cardinal symptoms of the disease remain elusive. Here, we compared a n-3 long-chain (LC) PUFA dietary supplementation (n-3 supp) obtained from fatty fish with a n-3 PUFA precursor diet (n-3 bal) obtained from plant oils in the valproic acid (VPA, 450 mg/kg at E12.5) ASD mouse model starting from embryonic life, throughout lactation and until adulthood. Maternal and offspring behaviors were investigated as well as several VPA-induced ASD biological features: cerebellar Purkinje cell (PC) number, inflammatory markers, gut microbiota, and peripheral and brain PUFA composition. Developmental milestones were delayed in the n-3 supp group compared to the n-3 bal group in both sexes. Whatever the diet, VPA-exposed offspring did not show ASD characteristic alterations in social behavior, stereotypies, PC number, or gut microbiota dysbiosis while global activity, gait, peripheral and brain PUFA levels as well as cerebellar TNF-alpha levels were differentially altered by diet and treatment according to sex. The current study provides evidence of beneficial effects of n-3 PUFA based diets, including one without LCPUFAs, on preventing several behavioral and cellular symptoms related to ASD.

## Introduction

Autism Spectrum Disorder (ASD) is a neurodevelopmental disorder characterized by persistent deficits in communication and social interactions, and restricted repetitive behaviors, interests, or activities^1^. Imaging and post-mortem studies in ASD patients have identified the cerebellum as one of the major affected brain regions with reduction of its volume and decreased Purkinje cell (PC) number^2^. Similar findings were observed in both genetic and environmental animal models, where the cerebellar regions crus I and crus II involved in motor and cognitive functions were shown to be the most affected by PC loss, with sex differences^3–5^. Many comorbidities are also associated with ASD including inflammation, gastrointestinal and eating disorders^6,7^. There is currently no cure or preventive strategies for this disease other than symptomatologic. Polyunsaturated fatty acids (PUFAs) from the n-6 and n-3 families are found in large quantities in brain cell membranes and are crucial for brain function and development^8,9^. Linoleic acid (LA, n-6) and alpha-linolenic acid (ALA, n-3) are essential fatty acids (FA) that cannot be synthetized by mammals and need to be provided through the diet. LA and ALA are then metabolized into long-chain (LC) PUFA, arachidonic acid (AA, n-6) and eicosapentaenoic acid (EPA, n-3)-docosahexaenoic acid (DHA, n-3), respectively. However, as ALA is not sufficient to supply the brain in n-3 LCPUFAs, additional dietary source from fatty fish is necessary^10^. Low levels of n-3 PUFA have been reported in the blood and brain of patients diagnosed with ASD^11–15^. As PUFAs from maternal diet are crucial for the developing brain during the perinatal period^9^, insufficient n-3 PUFA dietary supply during this critical period leads to aberrant brain lipid composition, metabolism, and signaling pathways^16,17^. This, in turn may be associated with neurodevelopmental and psychiatric disorders^8,18,19^. Thus, n-3 PUFA dietary supplementation, during the perinatal period for prevention and at adulthood for correction of or prevention from ASD have been explored in clinical and animal settings^20–26^.

While several studies point to potential benefits of n-3 PUFA supplementation during the perinatal period, the exact contribution of n-3 PUFAs species (i.e., precursor *versus* long-chain) on neurodevelopmental disorders are not well defined. This, along with the conflicting results obtained in clinical ASD studies, prompt us to perform a large scale and a side-by-side comparison of two diets. The first is a balanced diet (n-3 bal) with ALA as the only n-3 PUFA source from plant oils. The second is a n-3 LCPUFA supplemented diet (n-3 supp) with DHA and EPA from fatty fish. Both diets were isocaloric and differed in their fatty acid composition. Comparisons were performed in the valproic acid (VPA) mouse model of ASD, in males and females analyzed separately. Indeed, environmental factors acting in utero are suspected to contribute to the etiology of ASD^27^. Among these, VPA exposure during pregnancy has been consistently shown to be a major factor associated with developmental defects increasing the risk to develop ASD^28^. The VPA model has proven to have strong construct, face, and more recently, predictive validity^29–31^. Injection of VPA to pregnant rodent females systematically induces ASD-related syndromes in the offspring such as impaired social interaction^32–34^, repetitive behavior^34^ and delayed motor development^35^ (see also our reviews on this subject^2,36^). In this line, and prior to the current study, we had recently performed a full-scale analysis of this ASD mouse model on a wide range of behaviors with molecular and cellular correlates from early postnatal age to adulthood. A special focus was made on motor behavior and cerebellar implications under a standard diet containing comparable LA/ALA ratio (5.3) than the diets used here (6.2)^3^. We showed major social deficits, stereotypies and cerebellar motor and gait disorders in the VPA ASD mouse model which were correlated with cerebellar regional PC loss in crus I and crus II. This allowed us to obtain a solid starting point to investigate n-3 LCPUFA supplementation effects on the ASD phenotype. Here, we extend our analysis to inflammation and gut microbiome, to determine their implication in dam and in male and female offspring behavioral, cellular, and metabolic responses to differential diets.

## Results

### Maternal behavior is not affected by either VPA treatment or diet

VPA-exposed juvenile mice produce aberrant patterns of isolation stress-induced ultrasonic vocalizations^37^. This may influence in turn maternal behavior, which is key to normal social and motor development of offspring^38^. Here, we assessed various parameters related to maternal behavior following pup separation in relation with treatment and diet (Suppl. Fig. S1). Two-way ANOVA analysis indicated no differences between experimental groups, regardless of the treatment or diet on maternal care, nesting and stress related behaviors. In summary, our results indicate that VPA treatment did not affect maternal behavior when mothers were fed either n-3 LCPUFA or its precursors.

### Developmental milestones of offspring are delayed by n-3 LCPUFA supplementation

**Figure 1:**
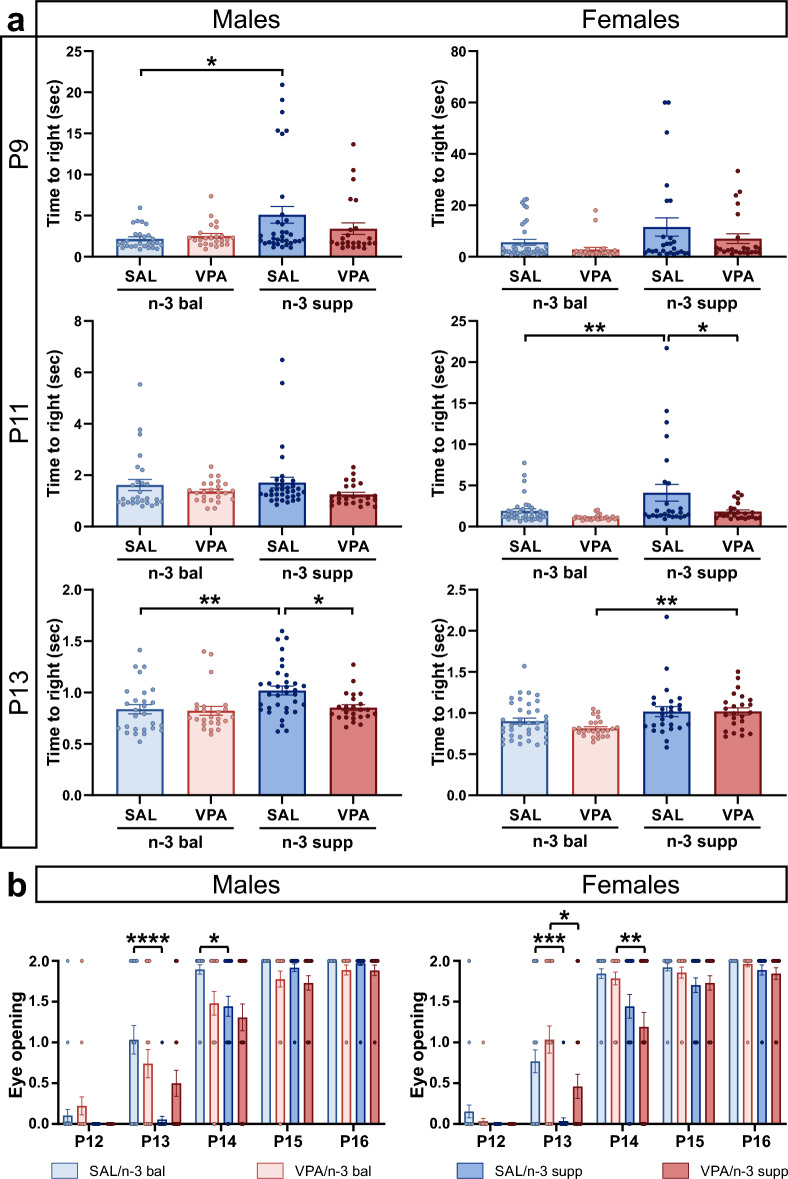
Developmental milestones of offspring are delayed by n-3 LCPUFA supplementation. (**a**) Righting reflex time at P9 (top), P11 (middle) and P13 (bottom) in both males (left) and females (right). n = 27 (SAL/n-3 bal male), 24 (VPA/n-3 bal male), 34 (SAL/n-3 supp male), 24 (VPA/n-3 supp male), 38 (SAL/n-3 bal female), 25 (VPA/n-3 bal female), 26 (SAL/n-3 supp female) and 24 (VPA/n-3 supp female) mice. (**b**) Eye opening score from P12 to P16 in both males (left) and females (right). n = 29 (SAL/n-3 bal male), 27 (VPA/n-3 bal male), 36 (SAL/n-3 supp male), 26 (VPA/n-3 supp male), 39 (SAL/n-3 bal female), 28 (VPA/n-3 bal female), 27 (SAL/n-3 supp female) and 26 (VPA/n-3 supp female) mice. Data are expressed as mean ± SEM and were analyzed through a two-way ANOVA followed by Tukey post-hoc multiple analysis. *p < 0.05, **p < 0.01, ***p < 0.001.

We have recently shown that prenatally VPA-exposed mice exhibit significant early postnatal behavioral impairments^3^. Here, we investigated righting reflex and eye-opening time-period from P9 to P16 in relation to treatment and diet (Fig. 1). SAL/n-3 supp males showed an aberrant righting reflex at P9 and P13 compared to both the SAL/n-3 bal group and compared to the VPA/n-3 supp group specifically at P13 only. Similar differences were found in females at P11 but not at P13 (Fig. 1a). In addition, eye opening was delayed in both male and female SAL/n-3 supp mice compared to SAL/n-3 bal mice at P13 and only in males at P14. Eye opening was also delayed in VPA/n-3 supp females compared to VPA/n-3 bal females at P13 and P14 (Fig. 1b).

These results indicate that n-3 supp diet affected sensorimotor development dependent on VPA treatment and sex of the animals.

### No major behavioral or cellular alterations following VPA treatment regardless of dietary intervention

**Figure 2:**
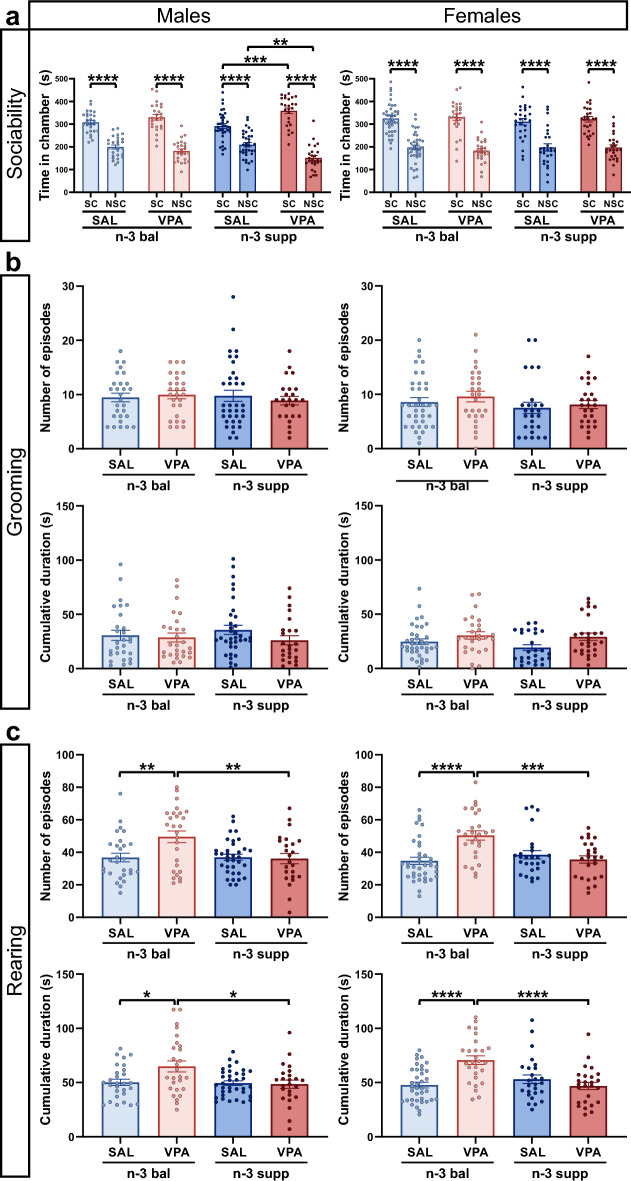
Offspring social, and grooming behavior are not altered by VPA following both n-3 PUFA diets. (**a**) Phase II from the 3-CT in both males (left) and females (right females). n = 27 (SAL/n-3 bal male), 25 (VPA/n-3 bal male), 35 (SAL/n-3 supp male), 25 (VPA/n-3 supp male), 39 (SAL/n-3 bal female), 27 (VPA/n-3 bal female), 27 (SAL/n-3 supp female) and 26 (VPA/n-3 supp female) mice. Data are expressed as mean ± SEM and were analyzed through a three-way ANOVA followed by Tukey post-hoc multiple analysis. *p < 0.05, **p < 0.01, ***p < 0.001. *SC* social chamber, *NSC* non-social chamber. (**b**) Number of grooming episodes and cumulative grooming duration in both males (left) and females (right). (**c**) Number of rearing episodes and cumulative rearing duration in both males (left) and females (right). n = 28 (SAL/n-3 bal male), 27 (VPA/n-3 bal male), 35 (SAL/n-3 supp male), 24 (VPA/n-3 supp male), 37 (SAL/n-3 bal female), 27 (VPA/n-3 bal female), 26 (SAL/n-3 supp female) and 26 (VPA/n-3 supp female) mice. Data are expressed as mean ± SEM and were analyzed through a three-way ANOVA for 3-CT or two-way ANOVA for grooming and rearing behavior, followed by Tukey post-hoc multiple analysis. *p < 0.05, **p < 0.01, ***p < 0.001.

Previous evidence from our group as well as other groups have reported that mice exposed prenatally to VPA and that were under a standard diet, consistently show reduced social behavior in the 3-CT test^3,29,30^. Here, using the same experimental procedures in the same housing conditions as previously, we report that both diets prevented the VPA-induced social impairments in male and female young adult mice (Fig. 2a). Three-way ANOVA showed that all groups spent more time in the social chamber compared to the nonsocial one. A treatment effect was found in VPA/n-3 supp males that were slightly more social than the SAL/n-3 supp group. We also investigated the time spent close to the novel mouse as well as the distance traveled in the chambers during this test and found the same outcome, where all groups showed normal social abilities (data not shown).

Stereotyped behavior has often been reported in various genetic and environmental animal models of ASD^2–5^. This behavior is reminiscent of repetitive behaviors in ASD patients and constitutes one of the major symptoms used in the diagnosis of ASD^1^. We have previously shown that prenatal exposure to VPA significantly increases stereotyped behavior in both males and females as compared to controls^3^. Here, we report no differences in grooming parameters between groups, (Fig. 2b). In addition, VPA/n-3 bal male and female mice showed an increased frequency and duration of rearing behavior compared to both SAL/n-3 bal and VPA/n-3 supp mice (Fig. 2c). However, VPA had no effect on rearing behavior in the supplemented groups as no differences were observed in VPA/n-3 supp *versus* SAL/n-3 supp groups or in SAL/n-3 bal *versus* SAL/n-3 supp groups.

**Figure 3:**
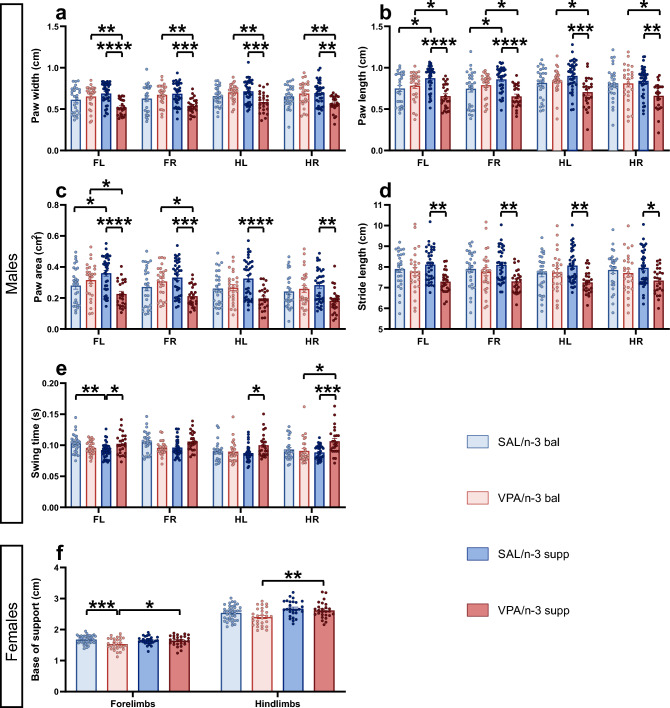
Offspring gait analysis exhibited sex differences in relation with diet. (**a**) Paw width in males. (**b**) Paw length in males. (**c**) Paw area in males. (**d**) Stride length in males. (**e**) Swing time in males. n = 27 (SAL/n-3 bal male), 26 (VPA/n-3 bal male), 36 (SAL/n-3 supp male), 24 (VPA/n-3 supp male) mice. (**f**) Fore and Hindlimbs Base of Support in females. n = 39 (SAL/n-3 bal female), 28 (VPA/n-3 bal female), 27 (SAL/n-3 supp female), 26 (VPA/n-3 supp female) mice. Data are expressed as mean ± SEM and were analyzed through a two-way ANOVA followed by Tukey post-hoc multiple analysis. *p < 0.05, **p < 0.01, ***p < 0.001.

Gait impairments have often been reported in both ASD patients and animal models, however they are not yet considered as a diagnostic criteria in ASD^39,40^. We have previously shown significant gait deficits in genetic and environmental ASD animal models, including the VPA model^2–5^. Here, we show moderate gait deficits, which were for the most part observed in the VPA/n-3 supp male group as compared to the SAL/n-3 supp group (Fig. 3). For instance, dynamic and temporal parameters such as stride length and swing time were affected in the supplemented groups under VPA. Morphological parameters such as paw width, paw length and paw area were also altered by both treatment and diet. In females, only a few parameters were affected, such as kinematic-related fore and hindlimbs base of support. VPA/n-3 bal females showed a decreased base of support compared to the VPA/n-3 supp group on both forelimbs and hindlimbs, and additionally to SAL/n-3 bal females for forelimbs only.

Taken together, these results indicate that both diets were able to reduce if not compensate for ASD related social, motor and gait symptoms, whatever the treatment, diet, or sex.

We have previously shown significant PC number decreases in the crus I or crus II cerebellar areas in both environmental and genetic ASD animal models^3–5^. Here, we examined PC number in crus I and crus II in all experimental groups (Suppl. Fig. S2). Two-way ANOVA analysis showed no treatment or diet effect in either males or females These results indicate that both n-3 bal and n-3 supp diets protected from PC loss induced by VPA in males and females alike, contrary to previous observations in mice fed with a standard animal facility diet.

### Diet, but not VPA treatment, differentially alters n-3 and n-6 LCPUFA levels in the liver, but not in the cerebellum

**Figure 4:**
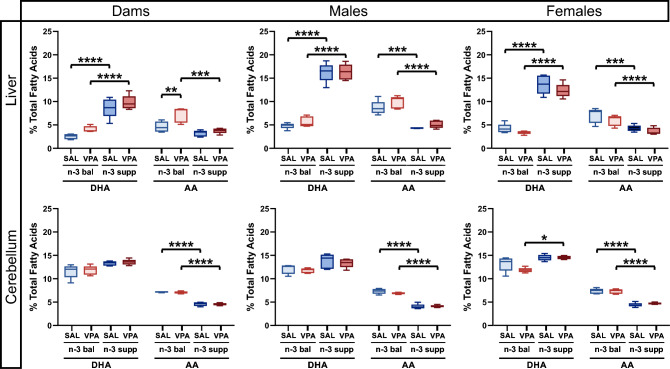
Both n-3 and n-6 LCPUFA profiles are modified in the liver, but not in the cerebellum, in relation with diet. Liver DHA and AA levels (top) in dams (left), offspring males (center) and females (right). n = 5 mice per group except 4 on SAL/n-3 supp male AA levels. Cerebellum DHA and AA levels (bottom) in dams (left), offspring males (center) and females (right). n = 5 mice per group except 4 on SAL/n-3 bal male and VPA/n-3 supp female AA levels. Data are expressed as median and min to max and were analyzed through a two-way ANOVA followed by Tukey post-hoc multiple analysis. *p < 0.05, **p < 0.01, ***p < 0.001.

We assessed the FA profile of the liver and the cerebellum, a cerebral region with high concentrations of DHA, sensitive to changes in FA composition, and that is at the crossroad of many cellular and molecular alterations in ASD, including loss of PC ^41^. Analyses were performed longitudinally to compare dam and offspring FA profiles. Given that n-3 LCPUFA supplementation was provided through diet, we analyzed, among others, levels of n-3 and n-6 LCPUFAs, DHA, EPA and AA (Fig. 4 and Suppl. Fig. S3). EPA levels were significantly increased in both the cerebellum and the liver in dams, male and female offspring, as expected. In the liver, in all n-3 LCPUFA supplemented groups, whether dams, male or female offspring, a significant increase (up to threefold) in DHA levels was found compared to n-3 balanced groups. In the cerebellum, DHA levels also increased in the VPA/n-3 supp group compared to VPA/n-3 bal animals, but only moderately (+ 25% approximately) and only in female offspring. Interestingly, no group differences in DHA cerebellar levels were found in dams and male offspring. The AA levels decreased in SAL/n-3 supp and VPA/n-3 supp groups compared to SAL/n-3 bal and VPA/n-3 bal groups respectively both in the liver and the cerebellum and in males and females. However, dam AA levels in the liver increased in the VPA/n-3 bal compared to both SAL/n-3 bal and VPA/n-3 supp.

These results indicate a major effect of n-3 LCPUFA supplementation on DHA and AA levels within the liver but not within the cerebellum where DHA levels were equivalent (saline and VPA males) or only slightly increased (in VPA females).

### Moderate to no alterations in inflammatory markers in conjunction with diet and treatment

**Figure 5:**
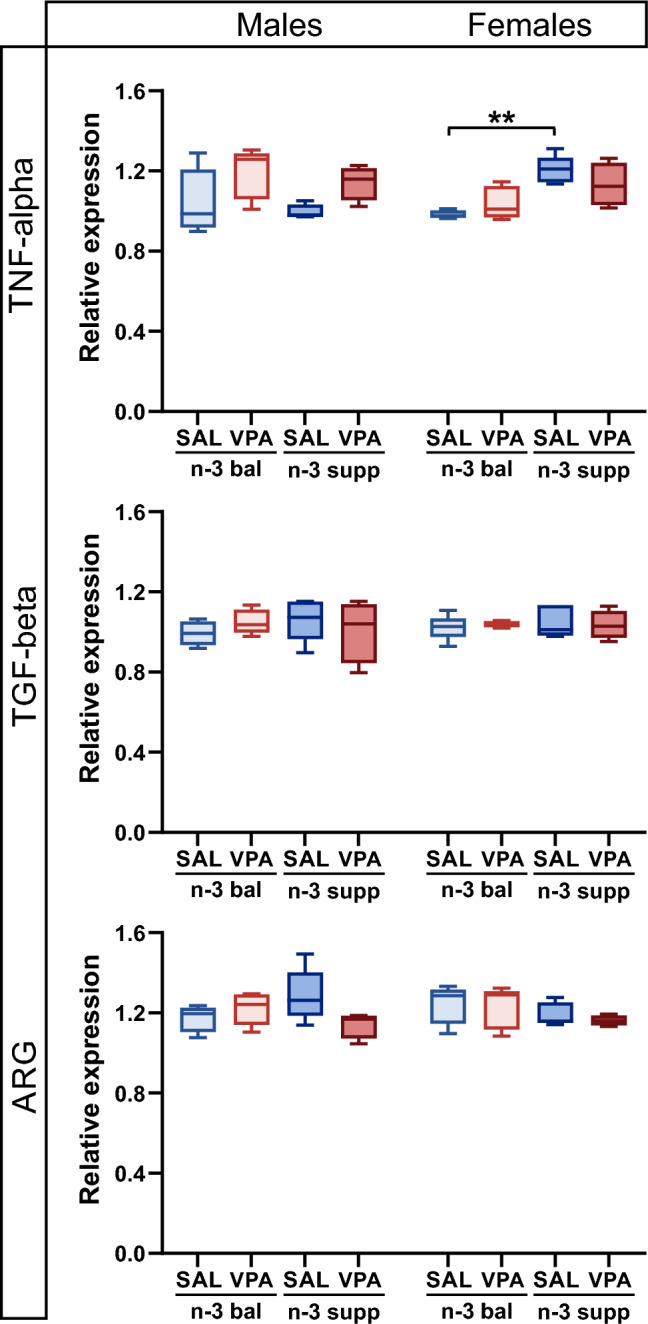
Moderate to no alteration in inflammatory markers in conjunction with diet and treatment. TNF-alpha cerebellar levels (top) in both males and females. n = 5 (SAL/n-3 bal male), 5 (VPA/n-3 bal male), 4 (SAL/n-3 supp male), 4 (VPA/n-3 supp male), 4 (SAL/n-3 bal female), 5 (VPA/n-3 bal female), 5 (SAL/n-3 supp female) and 5 (VPA/n-3 supp female) mice. TGF-beta cerebellar levels (middle) in both males and females. n = 5 (SAL/n-3 bal male), 5 (VPA/n-3 bal male), 5 (SAL/n-3 supp male), 4 (VPA/n-3 supp male), 5 (SAL/n-3 bal female), 5 (VPA/n-3 bal female), 5 (SAL/n-3 supp female), 4 (VPA/n-3 supp female) mice. ARG cerebellar levels (bottom) in both males and females. n = 5 (SAL/n-3 bal male), 5 (VPA/n-3 bal male), 5 (SAL/n-3 supp male), 4 (VPA/n-3 supp male), 5 (SAL/n-3 bal female), 5 (VPA/n-3 bal female), 5 (SAL/n-3 supp female), 4 (VPA/n-3 supp female) mice. Data are expressed as median and min to max and were analyzed through a two-way ANOVA followed by Tukey post-hoc multiple analysis. *p < 0.05, **p < 0.01, ***p < 0.001.

n-3 PUFAs are associated with anti-inflammatory processes and could influence behavior output^9^. Here, we investigated mRNA levels of several markers of inflammation in the offspring cerebellum: TNF-alpha, TGF-beta, and ARG. No differences were found between groups for TGF-beta and ARG, whatever the sex. However, TNF-alpha expression increased slightly but significantly in SAL/n-3 supp females compared to SAL/n-3 bal females. While VPA treatment increased TNF-alpha levels in males, no subsequent differences between groups were found (Fig. 5).

### Microbiota inter- and intra-diversity and bacteria relative abundance are not affected by diet

**Figure 6:**
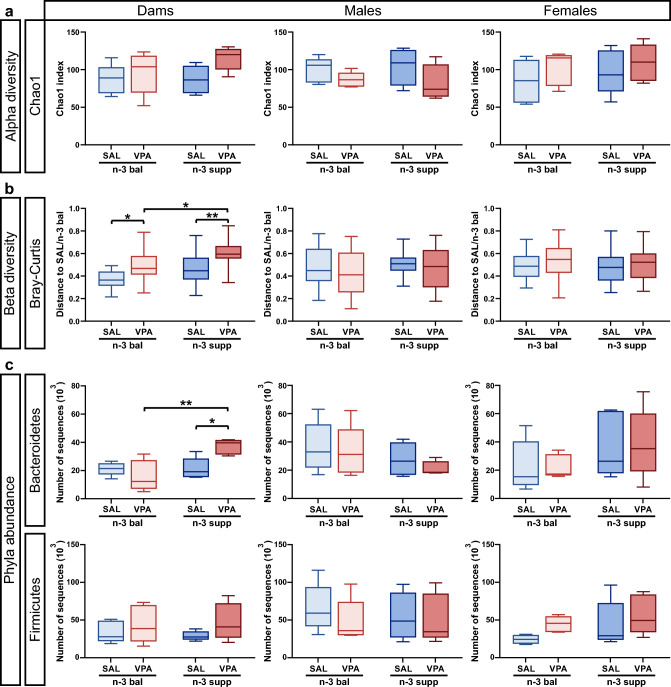
VPA does not alter offspring microbiota inter- and intra-diversity nor offspring microbiota relative abundance under both n-3 PUFA diets. Microbial diversity: (**a**) Chao1 index (first line) in dams (left), offspring males (center) and females (right). n = 5 mice per group. (**b**) Bray–Curtis index (third line) in dams (leftl), offspring males (center) and females (right). n = 10 (SAL/n-3 bal dam), 25 (VPA/n-3 bal dam), 25 (SAL/n-3 supp dam), 25 (VPA/n-3 supp dam), 10 (SAL/n-3 bal male), 25 (VPA/n-3 bal male), 20 (SAL/n-3 supp male), 25 (VPA/n-3 supp male), 10 (SAL/n-3 bal female), 25 (VPA/n-3 bal female), 25 (SAL/n-3 supp female) and 25 (VPA/n-3 supp female) mice. (**c**) Phyla abundance: Bacteroidetes abundance (first line) in dams (left), offspring males (center) and females (right). n = 5 mice per group except 4 in SAL/n-3 supp and VPA/n-3 supp male. Firmicutes abundance (second line) in dams (left), offspring males (center) and females (right). n = 5 mice per group except 4 in SAL/n-3 supp male and SAL/n-3 bal female. Data are expressed as median and min to max and were analyzed through a two-way ANOVA followed by Tukey post-hoc multiple analysis. *p < 0.05, **p < 0.01, ***p < 0.001.

Microbiota has been shown to be influenced by in utero VPA treatment on offspring and by n-3 PUFA supplementation or deficiency^42–44^. Using a n-3 balanced or n-3 LCPUFA supplemented diet, we found no differences in alpha-diversity in either dams or offspring. Interestingly, offspring beta-diversity was not different across groups, whereas dam beta-diversity was increased by VPA treatment, whatever the diet, with VPA n-3 suppl dams being the most dissimilar (Fig. 6a and Suppl. Figs. S4–S6). Additionally, in dams, but not in offspring, relative abundances of *Bacteroidetes* and *Actinobacteria* were increased in VPA/n-3 supp group compared to SAL/n-3 supp and VPA/n-3 bal group (Fig. 6b and Suppl. Fig. S7). No differences were found in female offspring phyla abundance, however, male offspring Bray–Curtis dissimilarity analysis showed that the VPA/n-3 supp group has a more diverse microbial composition compared to SAL/n-3 supp group (Fig. 6c and Suppl. Fig. S5). These results show no major differences between diets on the offspring microbiota, regardless of the treatment.

## Discussion

In this study, we investigated the potential benefits of adding n-3 LCPUFA as compared to an isocaloric balanced diet with a balanced LA (n-6)/ALA (n-3) ratio starting from the perinatal period to adulthood on a multiplicity of social, motor and gait behaviors as well as on cerebellar cellular, molecular, and metabolic correlates in both male and female VPA mice models of ASD. We found that adult VPA-exposed animals, regardless of the diet, were not showing social deficits, stereotypies or cerebellar PC loss, all of which are major hallmarks of ASD and were consistently reported in the VPA rodent model fed with a standard diet (see our reviews on the subject^2,36^). Developmental milestones, gait and inflammatory profiles were only slightly affected by diet in conjunction with VPA prenatal exposure. Gut microbiota composition was not altered either by diet or treatment. Additionally, cerebellar DHA levels were not affected by diet, while liver DHA levels dramatically increased with a n-3 LCPUFA supplementation. Using dams and their offspring, we were also able to perform longitudinal studies on several parameters. We found that while maternal behavior was not altered by either treatment or diet, dams exhibited a change in their microbiota composition and increased n-3 LCPUFA liver levels in conjunction with VPA treatment. Taken together, these results indicate that both diets with either n-3 PUFA precursors from plant oils or additional n-3 LCPUFAs from fatty fish provide protection against major behavioral, cellular, and molecular VPA-induced ASD symptoms.

In addition, we report that the n-3 LCPUFA supplemented groups exhibited a slight delay in both righting reflex and eye opening, in accordance with previous studies reporting adverse consequences in postnatal development after maternal dietary n-3 LCPUFA supplementation or deficiency during gestation and lactation^45–48^. Contrary to the common misconception that elevated levels of n-3 LCPUFAs are strictly beneficial, a change in the n-6/n-3 PUFA ratio, irrespective of its direction, was reported to be deleterious during development, with some consequences at adulthood, depending on the animal model^22,23^. Indeed, in both the BTBR and C57BL/6 J mouse strains, body weight was decreased following perinatal diet intervention whereas in the Fmr1 KO mouse, body weight was increased^22,23^. In addition, we report here that the n-3 balanced diet protected from VPA-induced developmental delays that we previously reported with a standard diet^3^, as both righting reflex and eye-opening scores in VPA-exposed animals were similar to the saline groups. Metabolic studies on this matter may help unravel the mechanisms behind this developmental delay.

Deficits in social preference or social novelty and increased grooming behavior are repeatedly found in several animal models of ASD including the VPA model under a standard diet, as we also reported recently^3–5^. Here, we found that both diets, n-3 PUFA balanced and n-3 LCPUFA supplemented, protected against social deficits and stereotypy anomalies, two major ASD-associated symptoms. To our knowledge, this study is the first to investigate n-3 LCPUFA and precursor effects on social behavior since the vast majority of studies compared n-3 LCPUFA supplementation and deficiency, where n-3 LCPUFA supplementation was shown to alleviate stereotypies and social behavior impairments^49^.

We have previously shown, in the same experimental settings, that rearing, which represents global activity, decreased in VPA-exposed animals fed with a regular animal facility diet^3^. Here, the n-3 balanced diet increased rearing behavior in VPA-exposed animals compared to controls and this was normalized with the n-3 LCPUFA supplemented diet. In physiological conditions, high n-3 LCPUFA supplementation for 3 weeks after weaning reduces rearing^50^, whereas in depression and anxiety models, which are ASD comorbidities, opposite results are found^51^.

Our previous work on several environmental and genetic ASD animal models fed with a regular diet consistently showed motor and gait impairments^3–5^. Gait is seldom explored in these models even though ASD patients exhibit an irregular walk and balance difficulties associated with cerebellar dysfunction^52^. Here, we showed that diet had a differential effect on ASD VPA male and female mice. The n-3 LCPUFA supplementation ameliorated gait parameters in females, with an increased hindlimb base of support, suggesting better stability, whereas VPA-exposed males with n-3 LCPUFA supplementation displayed dynamic, temporal and morphological impairments compared to controls, albeit of a lower magnitude than what we observed with animals under a regular diet^3^. These results suggest moderate sex-dependent gait deficiencies in conjunction with n-3 LCPUFA supplementation, possibly resulting from metabolic differences between males and females which are hypothesized to be estrogen-related^53^.

Crus I and crus II cerebellar regions are involved in both cognitive and motor functions, which make them a target of choice in ASD physiopathology^2,54^. A decrease in PC number has been widely reported in both ASD patients and in animal models, including the VPA mouse model under a standard diet^3,5,55–58^. Here, we showed that PC cell number in these regions was not altered between groups, regardless of the treatment, diet, or sex. These findings fit with our main hypothesis, which is that both diets, with n-3 PUFA precursors or with additional n-3 LCPUFAs, protect from VPA-induced ASD behavioral symptoms and cellular correlates.

We then investigated FA profiles in the liver and the cerebellum in order to determine whether n-3 PUFA dietary supplementation is associated with higher n-3 PUFA levels in these regions. The n-3 LCPUFA diet was highly supplemented in DHA and EPA, whereas the n-3 balanced diet contained n-3 precursors (ALA), with shared LA/ALA ratio of 6.2 in the two diets. Thus, our lipid analysis focused on n-3 and n-6 LCPUFAs, DHA, EPA and AA respectively. As expected, n-3 LCPUFA dietary supplementation resulted in a major decrease in liver and cerebellar AA levels and a significant increase in liver and cerebellar EPA levels in both males and females. However, liver DHA levels were increased in all groups. In the cerebellum, there was a diet effect with increased DHA levels in the VPA female group, but not in males. This indicates that a n-3 LCPUFA supplementation does not further increase cerebellar DHA levels in the male groups. These findings are in line with another study where a n-3 PUFA balanced diet with ALA as the only source of n-3 PUFA, increased DHA and decreased AA levels in the cortex and protected from deficits in emotional behavior in adult and old CD1 mice (2–5 months and 19–23 months), as compared to a n-3 deficient diet^59^. Previous studies have investigated the role of n-3 LCPUFA on inflammation and concluded that high DHA brain levels are linked to an anti-inflammatory profile whereas high AA brain levels are correlated with a pro-inflammatory profile^60–62^. We found that cerebellar TNF-alpha mRNA levels were increased in SAL/n-3 supp females only. This cytokine is one of the most studied and is known to increase levels of AMPA receptors and number of synapses, enhancing excitatory post-synaptic activity^63^. Through this mechanism, TNF-alpha has been found to improve learning and memory in rats^64^. Taken together, these results on PUFA liver and cerebellar profiles, and on cerebellar inflammation, highlight sex differences and female sensitization to n-3 LCPUFA supplementation.

ASD patients suffer from gastrointestinal issues (GI) hypothesized to result from a gut microbiota dysbiosis, i.e. a microbial composition imbalance^65,66^. Differences in alpha and beta-diversity and an imbalance in *Bacteroidetes* and *Firmicutes* have been consistently reported in the VPA ASD mouse model fed with a standard diet^42,67^. The n-3 LCPUFA supplementation was shown to contribute to microbiota diversity and homeostasis^44,68^, but studies on ALA effects are lacking. Here, we found no differences in alpha-diversity, beta-diversity, or phyla abundance in either male or female offspring, whatever the sex, treatment, or diet. This further consolidates our proposition that both diets, with or without n-3 LCPUFAs, bring behavioral and biological benefits in the VPA-induced ASD animal model.

One of the strengths of this study is the global and longitudinal approach where, in addition to male and female offspring analyzed separately, we also investigated treatment and diet influences on dam maternal behavior, liver and cerebellar FA profiles, as well as microbiota composition. Maternal care received by the pups during the first postnatal weeks can affect their behavior in adulthood^69,70^ and in our hands, we did not find any differences in maternal behavior between the dam groups, regardless of treatment or diet. These findings align with those of another study, where VPA treatment did not affect maternal behavior^71^. However, we found more drastic differences due to treatment and diet interaction in dams than in offspring pertaining to maternal FA profiles and gut microbiota composition. In fact, VPA-exposed dams with the n-3 balanced diet exhibited increased AA liver levels, which were normalized with the n-3 LCUPFA supplementation diet. In addition, *Bacteroidetes* and *Actinobacteria* proportion in VPA/n-3 supp dams increased, as did the beta-diversity in this experimental group. Dams may be more sensitive to diet changes as they were fed with a regular diet before gestation, whereas offspring were given the same diet, whether balanced or supplemented, from embryonic stage to sacrifice at adulthood.

There are few limitations to this study that need to be highlighted. (i), As shown in Supplementary Figs. S8 and S9, SAL/n-3 supp groups tend to have a higher body weight than the SAL/n-3 bal group. (ii), Further studies are needed with a direct comparison of VPA effects in mice under a standard diet or a n-3 deficient diet. (iii), Diets used in this study differed mainly by the presence or not of LCPUFA, and therefore they have a different fatty acid composition and origin (from plant oils or fatty fish), which could have also a biological impact.

Taken together, our findings indicate that n-3 PUFA dietary supplementation, with or without LCPUFAs, prevent ASD-related disturbances in the VPA mouse model. These beneficial effects were evidenced at the behavioral, cellular, and molecular levels, in both sexes, although females seem to be somewhat more sensitive to n-3 LCPUFA supplementation. Additional investigations are warranted, aiming at deciphering the underlying biological mechanisms of dietary effects on ASD symptoms. They also need to consider sex and age differences, two parameters seldomly investigated at least in preclinical settings, where experiments are performed mostly in young adult males under a regular diet.

## Methods

### Animals and treatment

Animal housing and experimental procedures were performed in accordance with the European Union directive (2010/63/EU) and validated by the ethics committee (Approval # 202002051628899). C57BL/6J mice (Charles River Laboratories) were housed in ventilated cages with access to food and water ad libitum. Room temperature was maintained at 23 °C on a 12-h-light/-dark cycle (08:00–20:00).

A total of 337 mice were used in this study: 55 females and 44 males were used for mating, resulting in 120 female and 118 male offspring. From the first gestational day (E0) and throughout gestation and nursing, pregnant females were fed with an isocaloric diet supplemented with n-3 LCPUFA (DHA and EPA) (n-3 supp) or not (n-3 bal) until weaning of their litter (SAFE, Augy, France). Both diets had a LA/ALA ratio of 6.2, and thus share the same amount of n-3 and n-6 precursors. As such, diets differed mostly by the presence of n-3 LCPUFA, DHA and EPA in the n-3 supp group (detailed description of diets are provided in Suppl. Tables S1 and S2). Animals under the n-3 supp diet received approximately 481.12 DHA and 703.99 EPA (mg/kg of body weight/day). Pregnant females received a single i.p. injection of either VPA (450 mg/kg, Sigma-Aldrich, P4543) or saline solution (NaCl 0.9%) at gestational day E12.5 as previously described^3,30^. After giving birth, dams were allocated to four experimental groups depending on prenatal treatment and diet: SAL fed with the n-3 bal diet (SAL/n-3 bal), VPA fed with n-3 bal diet (VPA/n-3 bal), SAL fed with the n-3 supp diet (SAL/n-3 supp) and VPA n-3 fed with the n-3 supp diet (VPA/n-3 supp) (timeline and experimental procedures are detailed in Suppl. Fig. S10). Male and female offspring were separated at weaning (postnatal day 28, P28) and housed by groups of 2 to 5 animals per cage, where they received ad libitum access to water and controlled access to the same diet as the respective dams. Dam body weight (postnatal day 14, P14) as well as male and female offspring body weights (weeks 4, 5, 6, 7 and 8) are presented in Supplementary Fig. S8. Male and female offspring food intakes are presented in Supplementary Fig. S9. Male and female offspring were affected into one of the following 8 groups for behavior analysis: 29 males and 39 females SAL/n-3 bal, 36 males and 27 females SAL/n-3 supp, 27 males and 28 females VPA/n-3 bal, and 26 males and 26 females VPA/n-3 supp. The same animals were used for gut microbiota, FA profile and inflammation markers. For this, 5 mice were randomly selected per group, with a maximum of 2 animals from the same litter and sex.

### Maternal and offspring behavior procedures

Maternal behavior experiments were performed on SAL/n-3 bal (n = 15), SAL/n-3 supp (n = 14), VPA/n-3 bal (n = 13), and VPA/n-3 supp (n = 12) groups. Dam performances on pup retrieval were assessed at P9. For this, both the litter and the dam were removed and separated from the home cage for 5 min before being placed back. Dam behavior directed towards the pups, or the environment was recorded for 15 min and analyzed on Solomon Coder (András Péter, Keele, UK).

Developmental milestones of offspring were assessed by measuring righting reflex at P9, P11 and P13 as well as eye opening from P12 to P16. Spontaneous activity in the cylinder was recorded at P48 for grooming and rearing scoring using Solomon Coder (András Péter, Keele, UK) as previously described^3,72^. Spatial, temporal, and kinematic gait parameters were analyzed during spontaneous walk at P49 using an automated gait analysis system (Gaitlab, Viewpoint, France) as previously described^3^. Social interaction was assessed between P50 and P60 using the three-chamber test (3-CT) as previously described^3,73^.

### Tissue processing, immunohistochemistry and stereology counting

At the end of behavioral experiments, mice were deeply anesthetized with pentobarbital (120 mg/kg) for tissue processing. Animal perfusion, brain retrieval, tissue sectioning and cresyl violet/neutral red staining were performed as previously described^4^. Stereological estimates of PC number within crus I and II cerebellar regions were obtained using the optical fractionator method (Mercator Software, Explora Nova, France) and systematic random sampling as previously described^4^ in the following groups: [Crus I: SAL/n-3 bal (males n = 11 and females n = 13), SAL/n-3 supp (males n = 11 and females n = 7), VPA/n-3 bal (males n = 10 and females n = 11) and VPA/n-3 supp (males n = 10 and females n = 9)] and [Crus II: SAL/n-3 bal (males n = 11 and females n = 12), SAL/n-3 supp (males n = 12 and females n = 9), VPA/n-3 bal (males n = 10 and females n = 11) and VPA/n-3 supp (males n = 10 and females n = 9].

### Fecal microbiome analysis

Fecal samples were collected from both the dam through lactation period (P15, P20 and P22) and the litter after weaning (P36, P41 and P43). Samples were conserved at − 80 °C in nucleic acid conservative buffer until DNA extraction (RNA Protect, Qiagen, Venlo, The Netherlands). Wet-lab and bioinformatic analysis were performed as previously described^74,75^. After verification on rarefaction curve, rarefaction (subsampling to 5000 sequences per sample) was performed before determination of alpha diversity metrics (Chao1′s and Shannon’s indexes) and beta diversity metrics (Bray–Curtis dissimilarity and weighted UniFrac^76^). ANCOM was used to identify differentially abundant genera among the groups^77^.

### Analysis of inflammation makers by qPCR

RNA expression of genes implicated in inflammatory process was investigated as previously described^78^. Briefly, RNA from hemi-cerebellum of dams and male and female offspring was extracted using TRIzol extraction kit (Invitrogen, Life Technologies, Saint-Quentin-Fallavier, France). Purity and concentration of RNA were determined using a Nanodrop 1000 spectrophotometer (Nanodrop technologies, Wilmington, DE, USA) and a bioanalyzer (Agilent, Les Ulis, France). Reverse transcription was performed on one or two micrograms of RNA by Superscript IV (Invitrogen, Life Technologies, Saint Aubin, France). TaqMan® specific primers were used to amplify genes of interest as previously described^78^ with a focus on tumor necrosis factor alpha (TNF-alpha, Mm00443258_m1), transforming growth factor-beta 1 (TGF-beta 1, Mm01178820_m1) and Arginase 1 (Arg1, Mm00475988_m1). The housekeeping gene was beta-2-microglobulin (B2M, Mm00437762_m1). Fluorescence was determined on a LightCycler® 480 instrument II (Roche, La Rochelle, France). Data were analyzed using the comparative threshold cycle (Ct) method and results were expressed as relative fold change to control target mRNA expression.

### Analysis of fatty acids in cerebellum and liver

FAs from the liver and the cerebellum were analyzed as previously described^79–81^. Briefly, liver and cerebellum lipids were extracted according to the Folch’s method^82^, FAs were transmethylated according to Morrison and Smith’s method^83^ and FA methyl esters (FAMEs) were analyzed on a FOCUS GC gas chromatograph (Thermo Electron Corporation) equipped with a split injector and a flame ionization detector, and a CPSil88-silica capillary column (100 m × 0.25 mm i.d., film thickness 0.20 μm, Varian, Les Ulis, France). The injector and the detector were maintained at 250 °C and 280 °C, respectively. Hydrogen was used as a carrier gas (inlet pressure 210 kPa). The oven temperature was fixed at 60 °C for 5 min, increased to 165 °C at a rate of 15 °C/min and then to 225 °C at a rate of 2 °C/min, and maintained at this temperature for 17 min. FAMEs were identified by making a comparison with commercial standards. FA composition is expressed as the percentage of total FAs.

### Statistical analysis

For experiments related to behavior, data are expressed as mean ± Standard Error of the Mean while cellular and metabolic data are expressed as median ± min to max. Data were analyzed using GraphPad Prism-9 software (La Jolla, California, USA). Outliers were identified by Grubbs’ test and were removed from subsequent statistical analysis (to a maximum of 1 outlier removed per outcome). Two-way or three-way analysis of variance (ANOVAs) were performed followed by Tukey post-hoc multiple comparisons test when appropriate. For all analyses, a p value < 0.05 was considered significant. Detailed statistical analysis on all groups is available in Supplementary Tables S3–S15. Other detailed statistical analysis and raw data are available upon reasonable request.

### ARRIVE guidelines

This study is in accordance with ARRIVE guidelines.

## Supplementary Information

Supplementary Information.

## Data Availability

Raw data for gut microbiota analysis are available on the following site (https://www.ncbi.nlm.nih.gov/bioproject/PRJNA854167). Accession SRA# are from SRR 19906240 to SRR19906299. Detailed statistical analysis is included in the supplementary material (Suppl. Tables S3–S15). Other details and raw data of all other experiments can be obtained upon reasonable request to the authors.
