# Supplementary material for: Mice prenatally exposed to valproic acid do not show autism-related disorders when fed with polyunsaturated fatty acid-enriched diets
Source: Sci Rep. 2023 Jul 11;13:11235. doi: 10.1038/s41598-023-38423-z (PMC10336048; doi:10.1038/s41598-023-38423-z)
Supplement: Supplementary file 1 — Supplementary Information. [file 41598_2023_38423_MOESM1_ESM.pdf]

Supplementary Table S1: Detailed composition of the n-3 supp diet and the n-3 bal diet.

	AIN modified with EPA/DHA (n-3 supp)	AIN modified without EPA/DHA (n-3 bal)
MERIGEL 100 CORN STARCH	400.0	400.0
CASEIN ACID	180.0	180.0
MALDEX 170 MALTODEXTRIN	140.0	140.0
SACCHAROSE	110.0	110.0
PRH 2502 without EPA/DHA	70.0	70.0
PRH 2502 with EPA/DHA	-	-
CELLULOSE	50.0	50.0
PM AIN 93M/G 3,5%	35.0	35.0
PV AIN 93M/G 1%	10.0	10.0
L-CYSTINE	3.0	3.0
CHOLINE CHLORIDE [>98%]	2.0	2.0
Protein %	16.0	16.0
Fat %	7.4	6.0
Minerals %	2.7	2.7
Cellulose %	3.6	3.6
Starch %	47.9	47.9
Sugars %	13.8	13.8
NFE %	63.1	63.1
Cholesterol mg/kg	0.4	19.8
Active ingredient mg/kg	-	-
Energy	-	-
ATWATER Kcal/Kg	3825.9	3825.9
ATWATER MJ/kg	16.0	16.0
E from protein %	16.7	16.7
E from fat %	17.4	17.4
E from NFE %	65.9	65.9
-E from sugar %	14.5	14.4
-E from starch %	50.1	50.1
Glucose %	< 0.5	< 0.5
Sucrose %	12.0	12.0
Lactose %	< 0.5	< 0.5
Arginine mg/kg	6120.0	6120.0
Cystine mg/kg	3570.0	3570.0
Lysine mg/kg	13468.0	13468.0
Methionine mg/kg	4860.0	4860.0
Tryptophan mg/kg	1890.0	1 890,0
Glycine mg/kg	3060.0	3 060,0
Calcium mg in end product	4973.0	4973.0
Phosphorus mg in end product	2616.0	2616.0
Sodium mg in end product	1438.0	1438.0
Potassium mg in end product	3691.0	3691.0
Magnesium mg in end product	642.0	642.0
Manganese mg in end product	12.0	12.0
Iron mg in end product	55.0	55.0
Copper mg in end product	6.2	6.2
Zinc mg in end product	42.0	42.0
Chlorine mg in end product	1417.0	1417.0
Vitamin A IU in end product	4261.0	4261.0
Vitamin D3 IU in end product	1250.0	1250.0
Vitamin E IU in end product	95.0	95.0
Vitamin K3 mg in end product	6.1	6.1
Vitamin B1 mg in end product	6.0	6.0
Vitamin B2 mg in end product	5.8	5.8
Vitamin B3 mg in end product	34.0	34.0
Vitamin B5 mg in end product	16.0	16.0
Vitamin B6 mg in end product	7.0	7.0
Vitamin B9 mg in end product	2.0	2.0
Vitamin B12 mg in end product	0.025	0.025
Biotin mg in end product	0.2	0.2
Choline mg in end product	1485.0	1485.0

Supplementary Table S2: Detailed fatty acid profiles for the n-3 supp diet and the n-3 bal diet.

**FATTY ACID PROFILES (mg/kg)**	**AIN modified with EPA/DHA (n-3 supp)**	**AIN modified without EPA/DHA (n-3 bal)**
Saturated Fatty Acids (SFA)	*14764.8*	*24469.7*
C10:0 Capric acid (decanoic)	0.1	0.1
C12:0 Lauric acid (dodecanoic)	56.2	52.3
C14:0 Myristic acid (tetradecanoic)	3100.4	595.5
C15:0 Pentadecylic acid (pentadecanoic)	218.9	-
C16:0 Palmitic acid (hexadecanoic)	8609.9	18879.2
C17:0 Margaric acid (heptadecanoic)	197.9	-
C17:0 iso Margaric acid (isoheptadecanoic)	74.6	-
C17:0 anteiso 14-methyl-hexadecanoic acid	52.8	-
C18:0 Stearic acid (octodecanoic)	2231.8	4778.3
C20:0 Arachidic acid (eicosanoic)	149.3	164.3
C22:0 Behenic acid (docosanoic)	51.4	-
C24:0 Lignoceric acid (tetracosanoic)	21.4	-
Unsaturated Fatty Acids (UFA)	*45788.3*	*40754.9*
Monounsaturated Fatty Acids (MUFA)	*19294.3*	*29100.9*
C14:1 Myristoleic acid	17.7	112.0
C16:1 **n-7** Palmitoleic acid (9Z-hexadecenoic)	4102.0	742.3
C17:1 anteiso 14-methyl-8-hexadecenoic acid	131.0	-
C17:1 Heptadecenoic acid	65.5	-
C18:1 **n-9** (FFA /AGL) Oleic acid and isomers (9Z-octadecenoic)	13755.0	28243.6
C19:1 Nonadecaneoic acid	73.3	-
C20:1 Gadoleic acid (eicosenic acid)	637.0	3.0
C22:1 **n-9** Erucic acid (13Z-docosaenoic)	318.5	-
C24:1 **n-9** Nervonic acid (15Z-tetracosaenoic)	194.3	-
Polyunsaturated Fatty Acids (PUFA)	*26494.0*	*11654.0*
C18:2 (LA) **n-6** Linoleic acid	9751.0	9726.5
C18:2 (CLA) **n-6** cj Conjugated linoleic acid	56.0	-
C18:3 (GLA) **n-6** Gamma-linolenic	94.2	-
C20:2 **n-6** Eicosadienoic acid	76.9	-
C20:3 (DGLA) **n-6** Dihomo-gamma-linolenic acid	213.9	-
C20:4 (AA) **n-6** Arachidonic acid	637.0	380.8
C22:4 **n-6** Docosatetraenoic acid	46.0	-
C22:5 (DPA n-6) **n-6** Docosapentaenoic acid	145.1	-
**n-6 Total**	**10964.0**	**10107.3**
C18:4 **n-3** Stearidonic acid	1274.0	-
C18:3 (ALA) **n-3** Alpha-linolenic acid	1570.1	1546.7
C20:3 (DALA) **n-3** Eicosatrienoic acid	42.3	-
C20:5 (EPA) **n-3** Eicosapentaenoic acid	7093.9	-
C22:5 (DPA n-3) **n-3** Docosapentaenoic acid	682.5	-
C22:6 (DHA) **n-3** Docosahexaenoic acid	4811.2	-
**n-3 Total**	**15474.0**	**1546.7**
**n-6/n-3 ratio**	**0.7**	**6.5**
**LA/ALA ratio**	**6.2**	**6.3**

Supplementary Table S3: Detailed statistics on grooming and rearing behavior for both male and female offspring.

		2-way ANOVA	F(1, 110)	p value	summary		
**Grooming Males**	Frequency	Interaction	0.6065	0.4378	*ns*		
		Nutrition	0.19	0.6638	*ns*		
		Treatment	0.0493	0.8247	*ns*		

		2-way ANOVA	F(1, 110)	p value	summary		
**Grooming Males**	Duration	Interaction	0.7574	0.386	*ns*		
		Nutrition	0.0768	0.7852	*ns*		
		Treatment	1.735	0.1905	*ns*		

		2-way ANOVA	F(1, 112)	p value	summary		
**Grooming Females**	Frequency	Interaction	0.0452	0.8319	*ns*		
		Nutrition	2.044	0.1556	*ns*		
		Treatment	0.8044	0.3717	*ns*		

		2-way ANOVA	F(1, 112)	p value	summary	Tukey multiple comparisons	p value	summary
**Grooming Females**	Duration	Interaction	0.4909	0.4850	*ns*	n-3 bal:SAL *vs* n-3 bal:VPA	0.4781	*ns*
		Nutrition	1.284	0.2633	*ns*	n-3 bal:SAL *vs* n-3 supp:SAL	0.5403	*ns*
		Treatment	6.971	0.0095	**	n-3 bal:SAL *vs* n-3 supp:VPA	0.6828	*ns*
						n-3 bal:VPA *vs* n-3 supp:SAL	0.0547	*ns*
						n-3 bal:VPA *vs* n-3 supp:VPA	0.9915	*ns*
						n-3 supp:VPA *vs* n-3 supp:VPA	0.1134	*ns*

		2-way ANOVA	F(1, 110)	p value	summary	Tukey multiple comparisons	p value	summary
**Rearing Males**	Frequency	Interaction	3.013	0.0158	*	n-3 bal:SAL *vs* n-3 bal:VPA	0.0089	**
		Nutrition	5.689	0.0188	*	n-3 bal:SAL *vs* n-3 supp:SAL	>0.9999	*ns*
		Treatment	4.607	0.034	*	n-3 bal:SAL *vs* n-3 supp:VPA	0.9985	*ns*
						n-3 bal:VPA *vs* n-3 supp:SAL	0.0061	**
						n-3 bal:VPA *vs* n-3 supp:VPA	0.008	**
						n-3 supp:VPA *vs* n-3 supp:VPA	0.9963	*ns*

		2-way ANOVA	F(1, 110)	p value	summary	Tukey multiple comparisons	p value	summary
**Rearing Males**	Duration	Interaction	5.037	0.0268	*	n-3 bal:SAL *vs* n-3 bal:VPA	0.0198	*
		Nutrition	5.99	0.016	*	n-3 bal:SAL *vs* n-3 supp:SAL	0.998	*ns*
		Treatment	3.876	0.0515	*ns*	n-3 bal:SAL *vs* n-3 supp:VPA	0.988	*ns*
						n-3 bal:VPA *vs* n-3 supp:SAL	0.008	**
						n-3 bal:VPA *vs* n-3 supp:VPA	0.0108	*
						n-3 supp:VPA *vs* n-3 supp:VPA	0.9973	*ns*

		2-way ANOVA	F(1, 112)	p value	summary	Tukey multiple comparisons	p value	summary
**Rearing Females**	Frequency	Interaction	14.2	0.0003	***	n-3 bal:SAL *vs* n-3 bal:VPA	<0.0001	****
		Nutrition	5.168	0.0249	*	n-3 bal:SAL *vs* n-3 supp:SAL	0.6968	*ns*
		Treatment	6.682	0.011	*	n-3 bal:SAL *vs* n-3 supp:VPA	0.9958	*ns*
						n-3 bal:VPA *vs* n-3 supp:SAL	0.0066	**
						n-3 bal:VPA *vs* n-3 supp:VPA	0.0004	***
						n-3 supp:VPA *vs* n-3 supp:VPA	0.857	*ns*

		2-way ANOVA	F(1, 112)	p value	summary	Tukey multiple comparisons	p value	summary
**Rearing Females**	Duration	Interaction	17.86	<0.0001	****	n-3 bal:SAL *vs* n-3 bal:VPA	<0.0001	****
		Nutrition	7.154	0.0086	**	n-3 bal:SAL *vs* n-3 supp:SAL	0.6689	*ns*
		Treatment	5.87	0.017	*	n-3 bal:SAL *vs* n-3 supp:VPA	0.9977	*ns*
						n-3 bal:VPA *vs* n-3 supp:SAL	0.0039	**
						n-3 bal:VPA *vs* n-3 supp:VPA	<0.0001	****
						n-3 supp:VPA *vs* n-3 supp:VPA	0.6152	*ns*

Supplementary Table S4: Detailed statistics on righting reflex for both male and female offspring.

		**2-way ANOVA**	F(1, 105)	p value	summary	**Tukey multiple comparisons**	p value	summary
**Righting reflex Males**	P9	Interaction	1.948	0.1658	*ns*	n-3 bal:SAL *vs* n-3 bal:VPA	0.9874	*ns*
		Nutrition	6.812	0.0104	*	n-3 bal:SAL *vs* n-3 supp:SAL	0.0171	*
		Treatment	0.839	0.3618	*ns*	n-3 bal:SAL *vs* n-3 supp:VPA	0.648	*ns*
						n-3 bal:VPA *vs* n-3 supp:SAL	0.0569	*ns*
						n-3 bal:VPA *vs* n-3 supp:VPA	0.8477	*ns*
						n-3 supp:VPA *vs* n-3 supp:VPA	0.3405	*ns*
		**2-way ANOVA**	F(1, 105)	p value	summary	**Tukey multiple comparisons**	p value	summary
	P11	Interaction	0.3757	0.5412	*ns*	n-3 bal:SAL *vs* n-3 bal:VPA	0.7748	*ns*
		Nutrition	0.0071	0.9331	*ns*	n-3 bal:SAL *vs* n-3 supp:SAL	0.9787	*ns*
		Treatment	3.994	0.0483	*	n-3 bal:SAL *vs* n-3 supp:VPA	0.4796	*ns*
						n-3 bal:VPA *vs* n-3 supp:SAL	0.5088	*ns*
						n-3 bal:VPA *vs* n-3 supp:VPA	0.966	*ns*
						n-3 supp:VPA *vs* n-3 supp:VPA	0.2363	*ns*
		**2-way ANOVA**	F(1, 105)	p value	summary	**Tukey multiple comparisons**	p value	summary
	P13	Interaction	3.215	0.0758	*ns*	n-3 bal:SAL *vs* n-3 bal:VPA	0.9937	*ns*
		Nutrition	3.293	0.0137	*	n-3 bal:SAL *vs* n-3 supp:SAL	0.0089	**
		Treatment	4.719	0.0321	*	n-3 bal:SAL *vs* n-3 supp:VPA	0.9956	*ns*
						n-3 bal:VPA *vs* n-3 supp:SAL	0.0052	**
						n-3 bal:VPA *vs* n-3 supp:VPA	0.9635	*ns*
						n-3 supp:VPA *vs* n-3 supp:VPA	0.0246	*
		**2-way ANOVA**	F(1, 109)	p value	summary	**Tukey multiple comparisons**	p value	summary
**Righting reflex Females**	P9	Interaction	0.1687	0.6821	*ns*	n-3 bal:SAL *vs* n-3 bal:VPA	0.7279	*ns*
		Nutrition	6.231	0.0141	*	n-3 bal:SAL *vs* n-3 supp:SAL	0.1338	*ns*
		Treatment	3.265	0.0735	*ns*	n-3 bal:SAL *vs* n-3 supp:VPA	0.9575	*ns*
						n-3 bal:VPA *vs* n-3 supp:SAL	0.0206	*
						n-3 bal:VPA *vs* n-3 supp:VPA	0.5044	*ns*
						n-3 supp:VPA *vs* n-3 supp:VPA	0.4417	*ns*
		**2-way ANOVA**	F(1, 109)	p value	summary	**Tukey multiple comparisons**	p value	summary
	P11	Interaction	2.109	0.1493	*ns*	n-3 bal:SAL *vs* n-3 bal:VPA	0.6546	*ns*
		Nutrition	8.033	0.0055	**	n-3 bal:SAL *vs* n-3 supp:SAL	0.009	**
		Treatment	9.063	0.0032	**	n-3 bal:SAL *vs* n-3 supp:VPA	0.9992	*ns*
						n-3 bal:VPA *vs* n-3 supp:SAL	0.0007	***
						n-3 bal:VPA *vs* n-3 supp:VPA	0.7914	*ns*
						n-3 supp:VPA *vs* n-3 supp:VPA	0.0166	*
		**2-way ANOVA**	F(1, 109)	p value	summary	**Tukey multiple comparisons**	p value	summary
	P13	Interaction	1.098	0.2971	*ns*	n-3 bal:SAL *vs* n-3 bal:VPA	0.4265	*ns*
		Nutrition	13.84	0.0003	***	n-3 bal:SAL *vs* n-3 supp:SAL	0.191	*ns*
		Treatment	1.008	0.3176	*ns*	n-3 bal:SAL *vs* n-3 supp:VPA	0.1968	*ns*
						n-3 bal:VPA *vs* n-3 supp:SAL	0.0088	**
						n-3 bal:VPA *vs* n-3 supp:VPA	0.0098	**
						n-3 supp:VPA *vs* n-3 supp:VPA	>0.9999	*ns*

Supplementary Table S5: Detailed statistics on eye opening for both male and female offspring.

		2-way ANOVA	F(1, 114)	p value	summary	Tukey multiple comparisons	p value	summary
**Eye opening Males**	P12	Interaction	0.8741	0.3518	*ns*	n-3 bal:SAL *vs* n-3 bal:VPA	0.5664	*ns*
		Nutrition	6.571	0.0117	*	n-3 bal:SAL *vs* n-3 supp:SAL	0.621	*ns*
		Treatment	0.8741	0.3518	*ns*	n-3 bal:SAL *vs* n-3 supp:VPA	0.6786	*ns*
						n-3 bal:VPA *vs* n-3 supp:SAL	0.0577	*ns*
						n-3 bal:VPA *vs* n-3 supp:VPA	0.0903	*ns*
						n-3 supp:VPA *vs* n-3 supp:VPA	>0.9999	*ns*
		**2-way ANOVA**	F(1, 114)	p value	summary	**Tukey multiple comparisons**	p value	summary
	P13	Interaction	6.987	0.0094	**	n-3 bal:SAL *vs* n-3 bal:VPA	0.4651	*ns*
		Nutrition	19.07	<0.0001	****	n-3 bal:SAL *vs* n-3 supp:SAL	<0.0001	****
		Treatment	0.2912	0.5905	*ns*	n-3 bal:SAL *vs* n-3 supp:VPA	0.0472	*
						n-3 bal:VPA *vs* n-3 supp:SAL	0.0028	**
						n-3 bal:VPA *vs* n-3 supp:VPA	0.6504	*ns*
						n-3 supp:VPA *vs* n-3 supp:VPA	0.1052	*ns*
		**2-way ANOVA**	F(1, 114)	p value	summary	**Tukey multiple comparisons**	p value	summary
	P14	Interaction	1.19	0.2776	*ns*	n-3 bal:SAL *vs* n-3 bal:VPA	0.1142	*ns*
		Nutrition	6.019	0.0157	*	n-3 bal:SAL *vs* n-3 supp:SAL	0.0465	*
		Treatment	4.679	0.0326	*	n-3 bal:SAL *vs* n-3 supp:VPA	0.0104	*
						n-3 bal:VPA *vs* n-3 supp:SAL	0.9966	*ns*
						n-3 bal:VPA *vs* n-3 supp:VPA	0.7941	*ns*
						n-3 supp:VPA *vs* n-3 supp:VPA	0.8665	*ns*
		**2-way ANOVA**	F(1, 114)	p value	summary	**Tukey multiple comparisons**	p value	summary
	P15	Interaction	0.07514	0.7845	*ns*	n-3 bal:SAL *vs* n-3 bal:VPA	0.0979	*ns*
		Nutrition	0.9674	0.3274	*ns*	n-3 bal:SAL *vs* n-3 supp:SAL	0.7859	*ns*
		Treatment	9.485	0.0026	**	n-3 bal:SAL *vs* n-3 supp:VPA	0.0308	*
						n-3 bal:VPA *vs* n-3 supp:SAL	0.4243	*ns*
						n-3 bal:VPA *vs* n-3 supp:VPA	0.9636	*ns*
						n-3 supp:VPA *vs* n-3 supp:VPA	0.1856	*ns*
		**2-way ANOVA**	F(1, 114)	p value	summary	**Tukey multiple comparisons**	p value	summary
	P16	Interaction	0.07265	0.788	*ns*	n-3 bal:SAL *vs* n-3 bal:VPA	0.2939	*ns*
		Nutrition	0.1351	0.7139	*ns*	n-3 bal:SAL *vs* n-3 supp:SAL	0.9647	*ns*
		Treatment	5.193	0.0245	*	n-3 bal:SAL *vs* n-3 supp:VPA	0.2701	*ns*
						n-3 bal:VPA *vs* n-3 supp:SAL	0.5062	*ns*
						n-3 bal:VPA *vs* n-3 supp:VPA	0.9999	*ns*
						n-3 supp:VPA *vs* n-3 supp:VPA	0.4718	*ns*

		2-way ANOVA	F(1, 116)	p value	summary	Tukey multiple comparisons	p value	summary
**Eye opening Females**	P12	Interaction	1.178	0.28	*ns*			
		Nutrition	3.033	0.0842	*ns*			
		Treatment	1.178	0.28	*ns*			
		**2-way ANOVA**	F(1, 116)	p value	summary	**Tukey multiple comparisons**	p value	summary
	P13	Interaction	0.3241	0.5703	*ns*	n-3 bal:SAL *vs* n-3 bal:VPA	0.481	*ns*
		Nutrition	22.15	<0.0001	****	n-3 bal:SAL *vs* n-3 supp:SAL	0.0009	***
		Treatment	6.296	0.0142	*	n-3 bal:SAL *vs* n-3 supp:VPA	0.3717	*ns*
						n-3 bal:VPA *vs* n-3 supp:SAL	<0.0001	****
						n-3 bal:VPA *vs* n-3 supp:VPA	0.0293	*
						n-3 supp:VPA *vs* n-3 supp:VPA	0.1729	*ns*
		**2-way ANOVA**	F(1, 116)	p value	summary	**Tukey multiple comparisons**	p value	summary
	P14	Interaction	0.7	0.4045	*ns*	n-3 bal:SAL *vs* n-3 bal:VPA	0.9792	*ns*
		Nutrition	18.86	<0.0001	****	n-3 bal:SAL *vs* n-3 supp:SAL	0.0521	*ns*
		Treatment	1.861	0.1751	*ns*	n-3 bal:SAL *vs* n-3 supp:VPA	0.0003	***
						n-3 bal:VPA *vs* n-3 supp:SAL	0.1784	*ns*
						n-3 bal:VPA *vs* n-3 supp:VPA	0.0034	**
						n-3 supp:VPA *vs* n-3 supp:VPA	0.4519	*ns*
		**2-way ANOVA**	F(1, 116)	p value	summary	**Tukey multiple comparisons**	p value	summary
	P15	Interaction	0.4327	0.512	*ns*	n-3 bal:SAL *vs* n-3 bal:VPA	0.8982	*ns*
		Nutrition	5.98	0.016	*	n-3 bal:SAL *vs* n-3 supp:SAL	0.1056	*ns*
		Treatment	0.07558	0.7839	*ns*	n-3 bal:SAL *vs* n-3 supp:VPA	0.1987	*ns*
						n-3 bal:VPA *vs* n-3 supp:SAL	0.4477	*ns*
						n-3 bal:VPA *vs* n-3 supp:VPA	0.6192	*ns*
						n-3 supp:VPA *vs* n-3 supp:VPA	0.994	*ns*
		**2-way ANOVA**	F(1, 116)	p value	summary	**Tukey multiple comparisons**	p value	summary
	P16	Interaction	0.005955	0.9386	*ns*	n-3 bal:SAL *vs* n-3 bal:VPA	0.936	*ns*
		Nutrition	6.349	0.0131	*	n-3 bal:SAL *vs* n-3 supp:SAL	0.2767	*ns*
		Treatment	0.7436	0.3903	*ns*	n-3 bal:SAL *vs* n-3 supp:VPA	0.0699	*ns*
						n-3 bal:VPA *vs* n-3 supp:SAL	0.6677	*ns*
						n-3 bal:VPA *vs* n-3 supp:VPA	0.2961	*ns*
						n-3 supp:VPA *vs* n-3 supp:VPA	0.9214	*ns*

Supplementary Table S6: Detailed statistics on three-chamber test (3CT) for both male and female offspring.

	3-way ANOVA	F(1, 216)	p value	summary	Tukey multiple comparisons	p value	summary
**3-CT Phase II Males**	Sociability	334.3	<0.0001	****	SC1:n-3 bal:SAL *vs* SC1:n-3 bal:VPA	0.8424	*ns*
	Treatment	0.08759	0.7676	*ns*	SC1:n-3 bal:SAL *vs* SC1:n-3 supp:SAL	0.9414	*ns*
	Nutrition	0.198	0.6568	*ns*	SC1:n-3 bal:SAL *vs* SC1:n-3 supp:VPA	0.0246	*
	Sociability x Treatment	1.245	0.2658	*ns*	SC1:n-3 bal:SAL *vs* NSC2:n-3 bal:SAL	<0.0001	****
	Sociability x Nutrition	30.53	<0.0001	****	SC1:n-3 bal:SAL *vs* NSC2:n-3 bal:VPA	<0.0001	****
	Treatment x Nutrition	0.02859	0.8659	*ns*	SC1:n-3 bal:SAL *vs* NSC2:n-3 supp:SAL	<0.0001	****
	Sociability x Treatment x Nutrition	8.172	0.0047	**	SC1:n-3 bal:SAL *vs* NSC2:n-3 supp:VPA	<0.0001	****
					SC1:n-3 bal:VPA *vs* SC1:n-3 supp:SAL	0.143	*ns*
					SC1:n-3 bal:VPA *vs* SC1:n-3 supp:VPA	0.5968	*ns*
					SC1:n-3 bal:VPA *vs* NSC2:n3 bal:SAL	<0.0001	****
					SC1:n-3 bal:VPA *vs* NSC2:n-3 bal:VPA	<0.0001	****
					SC1:n-3 bal:VPA *vs* NSC2:n-3 supp:SAL	<0.0001	****
					SC1:n-3 bal:VPA *vs* NSC2:n-3 supp:VPA	<0.0001	****
					SC1:n-3 supp:SAL *vs* SC1:n-3 supp:VPA	0.0002	***
					SC1:n-3 supp:SAL *vs* NSC2:n-3 bal:SAL	<0.0001	****
					SC1:n-3 supp:SAL *vs* NSC2:n-3 bal:VPA	<0.0001	****
					SC1:n-3 supp:SAL *vs* NSC2:n-3 supp:SAL	<0.0001	****
					SC1:n-3 supp:SAL *vs* NSC2:n-3 supp:VPA	<0.0001	****
					SC1:n-3 supp:VPA *vs* NSC2:n-3 bal:SAL	<0.0001	****
					SC1:n-3 supp:VPA *vs* NSC2:n-3 bal:VPA	<0.0001	****
					SC1:n-3 supp:VPA *vs* NSC2:n-3:SAL	<0.0001	****
					SC1:n-3 supp:VPA *vs* NSC2:n-3:VPA	<0.0001	****
					NSC2:n-3 bal:SAL *vs* NSC2:n-3 bal:VPA	0.9417	*ns*
					NSC2:n-3 bal:SAL *vs* NSC2:n-3 supp:SAL	0.9976	*ns*
					NSC2:n-3 bal:SAL *vs* NSC2:n-3 supp:VPA	0.0381	*
					NSC2:n-3 bal:VPA *vs* NSC2:n-3 supp:SAL	0.5593	*ns*
					NSC2:n-3 bal:VPA *vs* NSC2:n-3 supp:VPA	0.514	*ns*
					NSC2:n-3 supp:SAL *vs* NSC2:n-3 supp:VPA	0.0022	**

	3-way ANOVA	F(1, 112)	p value	summary	Tukey multiple comparisons	p value	summary
**3-CT Phase II Females**	Sociability	211,0000	<0.0001	****	SC1:n-3 bal:SAL vs SC1:n-3 bal:VPA	>0.9999	*ns*
	Treatment	0.07421	0.7855	*ns*	SC1:n-3 bal:SAL vs SC1:n-3 supp:SAL	0.9899	*ns*
	Nutrition	0.005719	0.9398	*ns*	SC1:n-3 bal:SAL vs SC1:n-3 supp:VPA	>0.9999	*ns*
	Sociability x Treatment	1.161	0.2824	*ns*	SC1:n-3 bal:SAL vs NSC2:n-3 bal:SAL	<0.0001	****
	Sociability x Nutrition	0.7393	0.3908	*ns*	SC1:n-3 bal:SAL vs NSC2:n-3 bal:VPA	<0.0001	****
	Treatment x Nutrition	0.2588	0.6114	*ns*	SC1:n-3 bal:SAL vs NSC2:n-3 supp:SAL	<0.0001	****
	Sociability x Treatment x Nutrition	0.05686	0.8117	*ns*	SC1:n-3 bal:SAL vs NSC2:n-3 supp:VPA	<0.0001	****
					SC1:n-3 bal:VPA vs SC1:n-3 supp:SAL	0.9555	*ns*
					SC1:n-3 bal:VPA vs SC1:n-3 supp:VPA	0.9996	*ns*
					SC1:n-3 bal:VPA vs NSC2:n3 bal:SAL	<0.0001	****
					SC1:n-3 bal:VPA vs NSC2:n-3 bal:VPA	<0.0001	****
					SC1:n-3 bal:VPA vs NSC2:n-3 supp:SAL	<0.0001	****
					SC1:n-3 bal:VPA vs NSC2:n-3 supp:VPA	<0.0001	****
					SC1:n-3 supp:SAL vs SC1:n-3 supp:VPA	0.9991	*ns*
					SC1:n-3 supp:SAL vs NSC2:n-3 bal:SAL	<0.0001	****
					SC1:n-3 supp:SAL vs NSC2:n-3 bal:VPA	<0.0001	****
					SC1:n-3 supp:SAL vs NSC2:n-3 supp:SAL	<0.0001	****
					SC1:n-3 supp:SAL vs NSC2:n-3 supp:VPA	<0.0001	****
					SC1:n-3 supp:VPA vs NSC2:n-3 bal:SAL	<0.0001	****
					SC1:n-3 supp:VPA vs NSC2:n-3 bal:VPA	<0.0001	****
					SC1:n-3 supp:VPA vs NSC2:n-3:SAL	<0.0001	****
					SC1:n-3 supp:VPA vs NSC2:n-3:VPA	<0.0001	****
					NSC2:n-3 bal:SAL vs NSC2:n-3 bal:VPA	0.9928	*ns*
					NSC2:n-3 bal:SAL vs NSC2:n-3 supp:SAL	>0.9999	*ns*
					NSC2:n-3 bal:SAL vs NSC2:n-3 supp:VPA	>0.9999	*ns*
					NSC2:n-3 bal:VPA vs NSC2:n-3 supp:SAL	0.9946	*ns*
					NSC2:n-3 bal:VPA vs NSC2:n-3 supp:VPA	0.9956	*ns*
					NSC2:n-3 supp:SAL vs NSC2:n-3 supp:VPA	>0.9999	*ns*

Supplementary Table S7: Detailed statistics on gait parameters: paw width and paw length for male offspring.

		2-way ANOVA	F(1, 109)	p value	summary	Tukey multiple comparisons	p value	summary
Paw Width Males	FL	Interaction	17.61	<0.0001	****	n-3 bal:SAL *vs* n-3 bal:VPA	0.6855	*ns*
		Nutrition	1.266	0.2631	*ns*	n-3 bal:SAL *vs* n-3 supp:SAL	0.1052	*ns*
		Treatment	6.745	0.0107	*	n-3 bal:SAL *vs* n-3 supp:VPA	0.0616	*ns*
						n-3 bal:VPA *vs* n-3 supp:SAL	0.6971	*ns*
						n-3 bal:VPA *vs* n-3 supp:VPA	0.0029	**
						n-3 supp:VPA *vs* n-3 supp:VPA	<0.0001	****
		**2-way ANOVA**	F(1, 109)	p value	summary	**Tukey multiple comparisons**	p value	summary
	FR	Interaction	13.84	0.0003	***	n-3 bal:SAL *vs* n-3 bal:VPA	0.6183	*ns*
		Nutrition	2.157	0.1448	*ns*	n-3 bal:SAL *vs* n-3 supp:SAL	0.3376	*ns*
		Treatment	3.861	0.052	*ns*	n-3 bal:SAL *vs* n-3 supp:VPA	0.0976	*ns*
						n-3 bal:VPA *vs* n-3 supp:SAL	0.983	*ns*
						n-3 bal:VPA *vs* n-3 supp:VPA	0.0039	**
						n-3 supp:VPA *vs* n-3 supp:VPA	0.0004	***
		**2-way ANOVA**	F(1, 109)	p value	summary	**Tukey multiple comparisons**	p value	summary
	HL	Interaction	14.25	0.0003	***	n-3 bal:SAL *vs* n-3 bal:VPA	0.5151	*ns*
		Nutrition	1.593	0.2096	*ns*	n-3 bal:SAL *vs* n-3 supp:SAL	0.2432	*ns*
		Treatment	3.184	0.0771	*ns*	n-3 bal:SAL *vs* n-3 supp:VPA	0.1704	*ns*
						n-3 bal:VPA *vs* n-3 supp:SAL	0.9803	*ns*
						n-3 bal:VPA *vs* n-3 supp:VPA	0.0054	**
						n-3 supp:VPA *vs* n-3 supp:VPA	0.0006	***
		**2-way ANOVA**	F(1, 109)	p value	summary	**Tukey multiple comparisons**	p value	summary
	HR	Interaction	10.43	0.016	**	n-3 bal:SAL *vs* n-3 bal:VPA	0.787	*ns*
		Nutrition	2.642	0.107	*ns*	n-3 bal:SAL *vs* n-3 supp:SAL	0.6288	*ns*
		Treatment	3.54	0.0626	*ns*	n-3 bal:SAL *vs* n-3 supp:VPA	0.0871	*ns*
						n-3 bal:VPA *vs* n-3 supp:SAL	0.9976	*ns*
						n-3 bal:VPA *vs* n-3 supp:VPA	0.0079	**
						n-3 supp:VPA *vs* n-3 supp:VPA	0.002	**

		2-way ANOVA	F(1, 109)	p value	summary	Tukey multiple comparisons	p value	summary
Paw Length Males	FL	Interaction	15.83	0.0001	***	n-3 bal:SAL *vs* n-3 bal:VPA	0.8851	*ns*
		Nutrition	0.002095	0.9636	*ns*	n-3 bal:SAL *vs* n-3 supp:SAL	0.0206	*
		Treatment	8.56	0.0042	**	n-3 bal:SAL *vs* n-3 supp:VPA	0.1884	*ns*
						n-3 bal:VPA *vs* n-3 supp:SAL	0.1501	*ns*
						n-3 bal:VPA *vs* n-3 supp:VPA	0.039	*
						n-3 supp:VPA *vs* n-3 supp:VPA	<0.0001	****
		**2-way ANOVA**	F(1, 109)	p value	summary	**Tukey multiple comparisons**	p value	summary
	FR	Interaction	15.45	0.0001	***	n-3 bal:SAL *vs* n-3 bal:VPA	0.7865	*ns*
		Nutrition	0.1344	0.7147	*ns*	n-3 bal:SAL *vs* n-3 supp:SAL	0.434	*
		Treatment	6.667	0.0111	*	n-3 bal:SAL *vs* n-3 supp:VPA	0.194	*ns*
						n-3 bal:VPA *vs* n-3 supp:SAL	0.3614	*ns*
						n-3 bal:VPA *vs* n-3 supp:VPA	0.0237	*
						n-3 supp:VPA *vs* n-3 supp:VPA	<0.0001	****
		**2-way ANOVA**	F(1, 109)	p value	summary	**Tukey multiple comparisons**	p value	summary
	HL	Interaction	10.88	0.0013	**	n-3 bal:SAL *vs* n-3 bal:VPA	0.9446	*ns*
		Nutrition	0.5899	0.4441	*ns*	n-3 bal:SAL *vs* n-3 supp:SAL	0.2377	*ns*
		Treatment	6.224	0.0141	*	n-3 bal:SAL *vs* n-3 supp:VPA	0.1259	*ns*
						n-3 bal:VPA *vs* n-3 supp:SAL	0.5794	*ns*
						n-3 bal:VPA *vs* n-3 supp:VPA	0.0362	*
						n-3 supp:VPA *vs* n-3 supp:VPA	0.0003	***
		**2-way ANOVA**	F(1, 109)	p value	summary	**Tukey multiple comparisons**	p value	summary
	HR	Interaction	5.953	0.0163	*	n-3 bal:SAL *vs* n-3 bal:VPA	>0.9999	*ns*
		Nutrition	2.628	0.1079	*ns*	n-3 bal:SAL *vs* n-3 supp:SAL	0.928	*ns*
		Treatment	6.072	0.0153	*	n-3 bal:SAL *vs* n-3 supp:VPA	0.0327	*
						n-3 bal:VPA *vs* n-3 supp:SAL	0.9243	*ns*
						n-3 bal:VPA *vs* n-3 supp:VPA	0.0365	*
						n-3 supp:VPA *vs* n-3 supp:VPA	0.0032	**

Supplementary Table S8: Detailed statistics on gait parameters: paw area and stride length for male offspring.

Parameter	Paw	2-way ANOVA	F(1, 109)	p value	summary	Tukey multiple comparisons	p value	summary
Paw Area Males	FL	Interaction	16.11	0.0001	***	n-3 bal:SAL *vs* n-3 bal:VPA	0.6514	*ns*
		Nutrition	0.05616	0.8131	*ns*	n-3 bal:SAL *vs* n-3 supp:SAL	0.0285	*
		Treatment	5.45	0.0214	*	n-3 bal:SAL *vs* n-3 supp:VPA	0.3057	*ns*
						n-3 bal:VPA *vs* n-3 supp:SAL	0.4107	*ns*
						n-3 bal:VPA *vs* n-3 supp:VPA	0.0259	*
						n-3 supp:VPA *vs* n-3 supp:VPA	<0.0001	****
	FR	Interaction	14.74	0.0002	***	n-3 bal:SAL *vs* n-3 bal:VPA	0.6077	*ns*
		Nutrition	0.6981	0.4059	*ns*	n-3 bal:SAL *vs* n-3 supp:SAL	0.1172	*ns*
		Treatment	4.246	0.0417	*	n-3 bal:SAL *vs* n-3 supp:VPA	0.2079	*ns*
						n-3 bal:VPA *vs* n-3 supp:SAL	0.8011	*ns*
						n-3 bal:VPA *vs* n-3 supp:VPA	0.0114	*
						n-3 supp:VPA *vs* n-3 supp:VPA	0.0002	***
	HL	Interaction	10.93	0.0013	**	n-3 bal:SAL *vs* n-3 bal:VPA	0.9972	*ns*
		Nutrition	0.01671	0.8974	*ns*	n-3 bal:SAL *vs* n-3 supp:SAL	0.088	*ns*
		Treatment	9.117	0.0032	**	n-3 bal:SAL *vs* n-3 supp:VPA	0.1482	*ns*
						n-3 bal:VPA *vs* n-3 supp:SAL	0.1478	*ns*
						n-3 bal:VPA *vs* n-3 supp:VPA	0.1021	*ns*
						n-3 supp:VPA *vs* n-3 supp:VPA	<0.0001	****
	HR	Interaction	8.022	0.0055	**	n-3 bal:SAL *vs* n-3 bal:VPA	0.9431	*ns*
		Nutrition	0.7613	0.3848	*ns*	n-3 bal:SAL *vs* n-3 supp:SAL	0.4619	*ns*
		Treatment	4.086	0.0457	*	n-3 bal:SAL *vs* n-3 supp:VPA	0.2082	*ns*
						n-3 bal:VPA *vs* n-3 supp:SAL	0.8305	*ns*
						n-3 bal:VPA *vs* n-3 supp:VPA	0.0669	*ns*
						n-3 supp:VPA *vs* n-3 supp:VPA	0.0036	**
Stride Length Males	FL	Interaction	4.862	0.0296	*	n-3 bal:SAL *vs* n-3 bal:VPA	0.9472	*ns*
		Nutrition	0.7517	0.3879	*ns*	n-3 bal:SAL *vs* n-3 supp:SAL	0.7495	*ns*
		Treatment	8.968	0.0034	**	n-3 bal:SAL *vs* n-3 supp:VPA	0.0486	*
						n-3 bal:VPA *vs* n-3 supp:SAL	0.398	*ns*
						n-3 bal:VPA *vs* n-3 supp:VPA	0.1709	*ns*
						n-3 supp:VPA *vs* n-3 supp:VPA	0.0016	**
	FR	Interaction	4.374	0.0388	*	n-3 bal:SAL *vs* n-3 bal:VPA	0.9373	*ns*
		Nutrition	0.7235	0.3969	*ns*	n-3 bal:SAL *vs* n-3 supp:SAL	0.79	*ns*
		Treatment	8.595	0.0041	**	n-3 bal:SAL *vs* n-3 supp:VPA	0.0556	*ns*
						n-3 bal:VPA *vs* n-3 supp:SAL	0.4178	*ns*
						n-3 bal:VPA *vs* n-3 supp:VPA	0.2025	*ns*
						n-3 supp:VPA *vs* n-3 supp:VPA	0.0024	**
	HL	Interaction	7.057	0.0091	**	n-3 bal:SAL *vs* n-3 bal:VPA	0.9984	*ns*
		Nutrition	0.1218	0.7278	*ns*	n-3 bal:SAL *vs* n-3 supp:SAL	0.3186	*ns*
		Treatment	5.841	0.0173	*	n-3 bal:SAL *vs* n-3 supp:VPA	0.2441	*ns*
						n-3 bal:VPA *vs* n-3 supp:SAL	0.4236	*ns*
						n-3 bal:VPA *vs* n-3 supp:VPA	0.01866	*ns*
						n-3 supp:VPA *vs* n-3 supp:VPA	0.0021	**
	HR	Interaction	1.98	0.1622	*ns*	n-3 bal:SAL *vs* n-3 bal:VPA	0.9241	*ns*
		Nutrition	0.6116	0.4359	*ns*	n-3 bal:SAL *vs* n-3 supp:SAL	0.988	*ns*
		Treatment	5.327	0.0229	*	n-3 bal:SAL *vs* n-3 supp:VPA	0.1607	*ns*
						n-3 bal:VPA *vs* n-3 supp:SAL	0.6727	*ns*
						n-3 bal:VPA *vs* n-3 supp:VPA	0.4578	*ns*
						n-3 supp:VPA *vs* n-3 supp:VPA	0.0413	*

Supplementary Table S9: Detailed statistics on gait parameters: swing time for male offspring and limbs base of support for female offspring.

		2-way ANOVA	F(1, 109)	p value	summary	Tukey multiple comparisons	p value	summary
**Swing Time Males**	FL	Interaction	11.54	0.001	***	n-3 bal:SAL *vs* n-3 bal:VPA	0.2111	*ns*
		Nutrition	0.8426	0.3607	*ns*	n-3 bal:SAL *vs* n-3 supp:SAL	0.0089	**
		Treatment	0.3286	0.5677	*ns*	n-3 bal:SAL *vs* n-3 supp:VPA	0.9955	*ns*
						n-3 bal:VPA *vs* n-3 supp:SAL	0.6885	*ns*
						n-3 bal:VPA *vs* n-3 supp:VPA	0.346	*ns*
						n-3 supp:VPA *vs* n-3 supp:VPA	0.0252	*
		**2-way ANOVA**	F(1, 109)	p value	summary	**Tukey multiple comparisons**	p value	summary
	FR	Interaction	11.66	0.0009	***	n-3 bal:SAL *vs* n-3 bal:VPA	0.0893	*ns*
		Nutrition	0.06713	0.796	*ns*	n-3 bal:SAL *vs* n-3 supp:SAL	0.0913	*ns*
		Treatment	0.00025	0.996	*ns*	n-3 bal:SAL *vs* n-3 supp:VPA	0.9982	*ns*
						n-3 bal:VPA *vs* n-3 supp:SAL	0.9973	*ns*
						n-3 bal:VPA *vs* n-3 supp:VPA	0.0704	*ns*
						n-3 supp:VPA *vs* n-3 supp:VPA	0.0717	*ns*
		**2-way ANOVA**	F(1, 109)	p value	summary	**Tukey multiple comparisons**	p value	summary
	HL	Interaction	5.048	0.0267	*	n-3 bal:SAL *vs* n-3 bal:VPA	0.9967	*ns*
		Nutrition	1.423	0.2356	*ns*	n-3 bal:SAL *vs* n-3 supp:SAL	0.8597	*ns*
		Treatment	3.771	0.0547	*ns*	n-3 bal:SAL *vs* n-3 supp:VPA	0.1511	*ns*
						n-3 bal:VPA *vs* n-3 supp:SAL	0.9452	*ns*
						n-3 bal:VPA *vs* n-3 supp:VPA	0.1014	*ns*
						n-3 supp:VPA *vs* n-3 supp:VPA	0.0161	*
		**2-way ANOVA**	F(1, 109)	p value	summary	**Tukey multiple comparisons**	p value	summary
	HR	Interaction	9.37	0.0028	**	n-3 bal:SAL *vs* n-3 bal:VPA	0.9621	*ns*
		Nutrition	2.746	0.1004	*ns*	n-3 bal:SAL *vs* n-3 supp:SAL	0.7205	*ns*
		Treatment	5.568	0.0201	*	n-3 bal:SAL *vs* n-3 supp:VPA	0.037	*
						n-3 bal:VPA *vs* n-3 supp:SAL	0.9542	*ns*
						n-3 bal:VPA *vs* n-3 supp:VPA	0.0104	*
						n-3 supp:VPA *vs* n-3 supp:VPA	0.0009	***
		**2-way ANOVA**	F(1, 111)	p value	summary	**Tukey multiple comparisons**	p value	summary
**Base of Support Females**	Forelimbs	Interaction	7.926	0.0058	**	n-3 bal:SAL *vs* n-3 bal:VPA	0.0009	***
		Nutrition	3.01	0.0855	*ns*	n-3 bal:SAL *vs* n-3 supp:SAL	0.8514	*ns*
		Treatment	5.937	0.0164	*	n-3 bal:SAL *vs* n-3 supp:VPA	0.9576	*ns*
						n-3 bal:VPA *vs* n-3 supp:SAL	0.0233	*
						n-3 bal:VPA *vs* n-3 supp:VPA	0.0144	*
						n-3 supp:VPA *vs* n-3 supp:VPA	0.9942	*ns*
		**2-way ANOVA**	F(1, 111)	p value	summary	**Tukey multiple comparisons**	p value	summary
	Hindlimbs	Interaction	0.9321	0.3364	*ns*	n-3 bal:SAL *vs* n-3 bal:VPA	0.1403	*ns*
		Nutrition	16.11	<0.0001	****	n-3 bal:SAL *vs* n-3 supp:SAL	0.1028	*ns*
		Treatment	3.73	0.056	*ns*	n-3 bal:SAL *vs* n-3 supp:VPA	0.4227	*ns*
						n-3 bal:VPA *vs* n-3 supp:SAL	0.0004	***
						n-3 bal:VPA *vs* n-3 supp:VPA	0.0054	**
						n-3 supp:VPA *vs* n-3 supp:VPA	0.9152	*ns*

Supplementary Table S10: Detailed statistics on liver and cerebellum DHA levels for male and female offspring and dams.

		2-way ANOVA	F (1, 16)	p value	summary	Tukey multiple comparisons	p value	summary
DHA levels Males	Liver	Interaction	0.3179	0.5807	*ns*	n-3 bal:SAL *vs* n-3 bal:VPA	0.8114	*ns*
		Nutrition	280.4	0.0001	****	n-3 bal:SAL *vs* n-3 supp:SAL	<0.0001	****
		Treatment	0.4777	0.4994	*ns*	n-3 bal:SAL *vs* n-3 supp:VPA	<0.0001	****
						n-3 bal:VPA *vs* n-3 supp:SAL	<0.0001	****
						n-3 bal:VPA *vs* n-3 supp:VPA	<0.0001	****
						n-3 supp:VPA *vs* n-3 supp:VPA	0.9997	*ns*

		2-way ANOVA	F (1, 16)	p value	summary	Tukey multiple comparisons	p value	summary
DHA levels Males	Cerebellum	Interaction	0.01774	0.8957	*ns*	n-3 bal:SAL *vs* n-3 bal:VPA	0.9662	*ns*
		Nutrition	11.99	0.0032	**	n-3 bal:SAL *vs* n-3 supp:SAL	0.0907	*ns*
		Treatment	0.6189	0.4429	*ns*	n-3 bal:SAL *vs* n-3 supp:VPA	0.2698	*ns*
						n-3 bal:VPA *vs* n-3 supp:SAL	0.0378	*
						n-3 bal:VPA *vs* n-3 supp:VPA	0.1269	*ns*
						n-3 supp:VPA *vs* n-3 supp:VPA	0.9138	*ns*

		2-way ANOVA	F (1, 16)	p value	summary	Tukey multiple comparisons	p value	summary
DHA levels Females	Liver	Interaction	0.1557	0.6984	*ns*	n-3 bal:SAL *vs* n-3 bal:VPA	0.7584	*ns*
		Nutrition	229.9	<0.0001	****	n-3 bal:SAL *vs* n-3 supp:SAL	<0.0001	****
		Treatment	3.209	0.0922	*ns*	n-3 bal:SAL *vs* n-3 supp:VPA	<0.0001	****
						n-3 bal:VPA *vs* n-3 supp:SAL	<0.0001	****
						n-3 bal:VPA *vs* n-3 supp:VPA	<0.0001	****
						n-3 supp:VPA *vs* n-3 supp:VPA	0.4352	*ns*

		2-way ANOVA	F (1, 15)	p value	summary	Tukey multiple comparisons	p value	summary
DHA levels Females	Cerebellum	Interaction	2.499	0.1348	*ns*	n-3 bal:SAL *vs* n-3 bal:VPA	0.1521	*ns*
		Nutrition	22.31	0.0003	***	n-3 bal:SAL *vs* n-3 supp:SAL	0.1445	*ns*
		Treatment	2.311	0.1493	**ns**	n-3 bal:SAL *vs* n-3 supp:VPA	0.1677	*ns*
						n-3 bal:VPA *vs* n-3 supp:SAL	0.0019	**
						n-3 bal:VPA *vs* n-3 supp:VPA	0.003	**
						n-3 supp:VPA *vs* n-3 supp:VPA	>0.9999	*ns*

		2-way ANOVA	F (1, 16)	p value	summary	Tukey multiple comparisons	p value	summary
DHA levels Dams	Liver	Interaction	0.1296	0.7235	*ns*	n-3 bal:SAL *vs* n-3 bal:VPA	0.3166	*ns*
		Nutrition	92.93	<0.0001	****	n-3 bal:SAL *vs* n-3 supp:SAL	<0.0001	****
		Treatment	4.674	0.0461	*	n-3 bal:SAL *vs* n-3 supp:VPA	<0.0001	****
						n-3 bal:VPA *vs* n-3 supp:SAL	0.0004	***
						n-3 bal:VPA *vs* n-3 supp:VPA	<0.0001	****
						n-3 supp:VPA *vs* n-3 supp:VPA	0.5914	*ns*

		2-way ANOVA	F (1, 16)	p value	summary	Tukey multiple comparisons	p value	summary
DHA levels Dams	Cerebellum	Interaction	0.003901	0.951	*ns*	n-3 bal:SAL *vs* n-3 bal:VPA	0.9871	*ns*
		Nutrition	15.66	0.0011	**	n-3 bal:SAL *vs* n-3 supp:SAL	0.0612	*ns*
		Treatment	0.2806	0.6036	*ns*	n-3 bal:SAL *vs* n-3 supp:VPA	0.0272	*
						n-3 bal:VPA *vs* n-3 supp:SAL	0.1123	*ns*
						n-3 bal:VPA *vs* n-3 supp:VPA	0.0518	*ns*
						n-3 supp:VPA *vs* n-3 supp:VPA	0.9745	*ns*

Supplementary Table S11: Detailed statistics on liver and cerebellum AA levels for male and female offspring and dams.

		2-way ANOVA	F (1, 15)	p value	summary	Tukey multiple comparisons	p value	summary
AA levels Males	Liver	Interaction	0.2263	0.6411	*ns*	n-3 bal:SAL *vs* n-3 bal:VPA	0.3198	*ns*
		Nutrition	81.87	<0.0001	****	n-3 bal:SAL *vs* n-3 supp:SAL	0.0002	***
		Treatment	3.872	0.0679	*ns*	n-3 bal:SAL *vs* n-3 supp:VPA	0.0006	***
						n-3 bal:VPA *vs* n-3 supp:SAL	<0.0001	****
						n-3 bal:VPA *vs* n-3 supp:VPA	<0.0001	****
						n-3 supp:VPA *vs* n-3 supp:VPA	0.7376	*ns*
		**2-way ANOVA**	F (1, 15)	p value	summary	**Tukey multiple comparisons**	p value	summary
	Cerebellum	Interaction	1.472	0.2439	*ns*	n-3 bal:SAL *vs* n-3 bal:VPA	0.498	*ns*
		Nutrition	237.3	<0.0001	****	n-3 bal:SAL *vs* n-3 supp:SAL	<0.0001	****
		Treatment	0.7667	0.395	*ns*	n-3 bal:SAL *vs* n-3 supp:VPA	<0.0001	****
						n-3 bal:VPA *vs* n-3 supp:SAL	<0.0001	****
						n-3 bal:VPA *vs* n-3 supp:VPA	<0.0001	****
						n-3 supp:VPA *vs* n-3 supp:VPA	0.9945	*ns*
		**2-way ANOVA**	F (1, 16)	p value	summary	**Tukey multiple comparisons**	p value	summary
AA levels Females	Liver	Interaction	0.2013	0.6597	*ns*	n-3 bal:SAL *vs* n-3 bal:VPA	0.4414	*ns*
		Nutrition	26.71	<0.0001	****	n-3 bal:SAL *vs* n-3 supp:SAL	0.0054	**
		Treatment	2.963	0.1045	*ns*	n-3 bal:SAL *vs* n-3 supp:VPA	0.0009	***
						n-3 bal:VPA *vs* n-3 supp:SAL	0.1098	*ns*
						n-3 bal:VPA *vs* n-3 supp:VPA	0.0196	*
						n-3 supp:VPA *vs* n-3 supp:VPA	0.8051	*ns*
		**2-way ANOVA**	F (1, 16)	p value	summary	**Tukey multiple comparisons**	p value	summary
	Cerebellum	Interaction	0.7189	0.409	*ns*	n-3 bal:SAL *vs* n-3 bal:VPA	0.9977	*ns*
		Nutrition	191.7	<0.0001	****	n-3 bal:SAL *vs* n-3 supp:SAL	<0.0001	****
		Treatment	0.3464	0.5644	**ns**	n-3 bal:SAL *vs* n-3 supp:VPA	<0.0001	****
						n-3 bal:VPA *vs* n-3 supp:SAL	<0.0001	****
						n-3 bal:VPA *vs* n-3 supp:VPA	<0.0001	****
						n-3 supp:VPA *vs* n-3 supp:VPA	0.7428	*ns*
		**2-way ANOVA**	F (1, 16)	p value	summary	**Tukey multiple comparisons**	p value	summary
AA levels Dams	Liver	Interaction	4.628	0.0471	*	n-3 bal:SAL *vs* n-3 bal:VPA	0.0065	**
		Nutrition	25.97	0.0001	***	n-3 bal:SAL *vs* n-3 supp:SAL	0.2007	*ns*
		Treatment	11.16	0.0042	**	n-3 bal:SAL *vs* n-3 supp:VPA	0.6106	*ns*
						n-3 bal:VPA *vs* n-3 supp:SAL	0.0001	***
						n-3 bal:VPA *vs* n-3 supp:VPA	0.0005	***
						n-3 supp:VPA *vs* n-3 supp:VPA	0.8343	*ns*
		**2-way ANOVA**	F (1, 16)	p value	summary	**Tukey multiple comparisons**	p value	summary
	Cerebellum	Interaction	0.1091	0.7454	*ns*	n-3 bal:SAL *vs* n-3 bal:VPA	0.9102	*ns*
		Nutrition	419.8	<0.0001	****	n-3 bal:SAL *vs* n-3 supp:SAL	<0.0001	****
		Treatment	0.3652	0.5541	*ns*	n-3 bal:SAL *vs* n-3 supp:VPA	<0.0001	****
						n-3 bal:VPA *vs* n-3 supp:SAL	<0.0001	****
						n-3 bal:VPA *vs* n-3 supp:VPA	<0.0001	****
						n-3 supp:VPA *vs* n-3 supp:VPA	0.9973	*ns*

Supplementary Table S12: Detailed statistics on cerebellar inflammatory markers for male and female offspring.

	2-way ANOVA	F (1, 14)	p value	summary	Tukey multiple comparisons	p value	summary
**TNF-alpha Males**	Interaction	0.001257	0.9722	*ns*	n-3 bal:SAL *vs* n-3 bal:VPA	0.2579	*ns*
	Nutrition	0.8143	0.3821	*ns*	n-3 bal:SAL *vs* n-3 supp:SAL	0.9091	*ns*
	Treatment	6.846	0.0203	*	n-3 bal:SAL *vs* n-3 supp:VPA	0.6296	*ns*
					n-3 bal:VPA *vs* n-3 supp:SAL	0.1055	*ns*
					n-3 bal:VPA *vs* n-3 supp:VPA	0.9262	*ns*
					n-3 supp:VPA *vs* n-3 supp:VPA	0.3231	*ns*

	2-way ANOVA	F (1, 14)	p value	summary	Tukey multiple comparisons	p value	summary
**TNF-alpha Females**	Interaction	3.272	0.092	*ns*	n-3 bal:SAL *vs* n-3 bal:VPA	0.6924	*ns*
	Nutrition	18.82	0.0007	***	n-3 bal:SAL *vs* n-3 supp:SAL	0.0033	**
	Treatment	0.06055	0.8092	*ns*	n-3 bal:SAL *vs* n-3 supp:VPA	0.0671	*ns*
					n-3 bal:VPA *vs* n-3 supp:SAL	0.0185	*
					n-3 bal:VPA *vs* n-3 supp:VPA	0.3189	*ns*
					n-3 supp:VPA *vs* n-3 supp:VPA	0.4894	*ns*

	2-way ANOVA	F (1, 15)	p value	summary
**TGF-beta Males**	Interaction	1.453	0.2467	*ns*
	Nutrition	0.0699	0.7951	*ns*
	Treatment	0.002308	0.9623	*ns*

	2-way ANOVA	F (1, 15)	p value	summary
**TGF-beta Females**	Interaction	0.276	0.607	*ns*
	Nutrition	0.1432	0.7104	*ns*
	Treatment	0.002345	0.962	*ns*

	2-way ANOVA	F (1, 15)	p value	summary	Tukey	p value	summary
**ARG Males**	Interaction	5.357	0.0352	*	n-3 bal:SAL *vs* n-3 bal:VPA	0.8209	*ns*
	Nutrition	0.1837	0.6743	*ns*	n-3 bal:SAL *vs* n-3 supp:SAL	0.2316	*ns*
	Treatment	1.262	0.279	*ns*	n-3 bal:SAL *vs* n-3 supp:VPA	0.9629	*ns*
					n-3 bal:VPA *vs* n-3 supp:SAL	0.6768	*ns*
					n-3 bal:VPA *vs* n-3 supp:VPA	0.5792	*ns*
					n-3 supp:VPA *vs* n-3 supp:VPA	0.1278	*ns*

	2-way ANOVA	F (1, 15)	p value	summary
**ARG Females**	Interaction	0.05062	0.825	*ns*
	Nutrition	2.418	0.1408	*ns*
	Treatment	0.3706	0.5518	*ns*

Supplementary Table S13: Detailed statistics on gut microbiota alpha-diveristy (Chao1) for male and female offspring and dams.

Chao1 Males	**2-way ANOVA**	F (1, 15)	p value	summary
	Interaction	0.222	0.6443	*ns*
	Nutrition	0.006715	0.9358	*ns*
	Treatment	3.81	0.0699	*ns*

Chao1 Females	**2-way ANOVA**	F (1, 16)	p value	summary
	Interaction	0.05185	0.8228	*ns*
	Nutrition	0.6942	0.417	*ns*
	Treatment	1.546	0.2317	*ns*

Chao1 Dams	**2-way ANOVA**	F (1, 16)	p value	summary
	Interaction	1.016	0.3285	*ns*
	Nutrition	1.067	0.3171	*ns*
	Treatment	4.006	0.0626	*ns*

Supplementary Table S14: Detailed statistics on gut microbiota beta-diveristy (Bray-Curtis) for male and female offspring and dams.

Group		2-way ANOVA	F	p value	summary	Tukey multiple comparisons	p value	summary
Bray-Curtis Males	n-3 bal:SAL	**2-way ANOVA**	F (1, 76)	p value	summary			
		Interaction	0.05298	0.8186	ns			
		Nutrition	1.1	0.2976	ns			
		Treatment	1.708	0.1952	ns			
	n-3 suppl:SAL	**2-way ANOVA**	F (1, 62)	p value	summary	**Tukey multiple comparisons**	p value	summary
		Interaction	4.934	0.03	*	n-3 bal:SAL *vs* n-3 bal:VPA	0.8139	ns
		Nutrition	1.014	0.3179	ns	n-3 bal:SAL *vs* n-3 supp:SAL	0.2177	ns
		Treatment	10.32	0.0021	**	n-3 bal:SAL *vs* n-3 supp:VPA	0.2132	ns
						n-3 bal:VPA *vs* n-3 supp:SAL	0.062	ns
						n-3 bal:VPA *vs* n-3 supp:VPA	0.7027	ns
						n-3 supp:VPA *vs* n-3 supp:VPA	0.0089	**
	n-3 bal:VPA	**2-way ANOVA**	F (1, 76)	p value	summary			
		Interaction	1.525	0.2207	ns			
		Nutrition	2.679	0.1058	ns			
		Treatment	1.149	0.2871	ns			
	n-3 supp:VPA	**2-way ANOVA**	F (1, 76)	p value	summary	**Tukey multiple comparisons**	p value	summary
		Interaction	0.002792	0.958	ns	n-3 bal:SAL *vs* n-3 bal:VPA	0.9549	ns
		Nutrition	5.968	0.0169	*	n-3 bal:SAL *vs* n-3 supp:SAL	0.199	ns
		Treatment	0.4386	0.5098	ns	n-3 bal:SAL *vs* n-3 supp:VPA	0.6654	ns
						n-3 bal:VPA *vs* n-3 supp:SAL	0.0709	ns
						n-3 bal:VPA *vs* n-3 supp:VPA	0.4239	ns
						n-3 supp:VPA *vs* n-3 supp:VPA	0.9708	ns
Bray-Curtis Females	n-3 bal:SAL	**2-way ANOVA**	F (1, 81)	p value	summary			
		Interaction	0.06632	0.7974	ns			
		Nutrition	0.1687	0.6824	ns			
		Treatment	0.553	0.4593	ns			
	n-3 suppl:SAL	**2-way ANOVA**	F (1, 81)	p value	summary			
		Interaction	1.606	0.2087	ns			
		Nutrition	1.293	0.2589	ns			
		Treatment	1.205	0.2756	ns			
	n-3 bal:VPA	**2-way ANOVA**	F (1, 81)	p value	summary			
		Interaction	0.02577	0.8729	ns			
		Nutrition	2.07	0.154	ns			
		Treatment	0.6494	0.4227	ns			
	n-3 supp:VPA	**2-way ANOVA**	F (1, 81)	p value	summary			
		Interaction	0.00559	0.9406	ns			
		Nutrition	0.3057	0.5819	ns			
		Treatment	1.254	0.2661	ns			
Bray-Curtis Dams	n-3 bal:SAL	**2-way ANOVA**	F (1, 81)	p value	summary	**Tukey multiple comparisons**	p value	summary
		Interaction	0.01057	0.9184	ns	n-3 bal:SAL *vs* n-3 bal:VPA	0.0324	*
		Nutrition	12.46	0.0007	***	n-3 bal:SAL *vs* n-3 supp:SAL	0.147	ns
		Treatment	20.78	<0.0001	****	n-3 bal:SAL *vs* n-3 supp:VPA	<0.0001	****
						n-3 bal:VPA *vs* n-3 supp:SAL	0.8287	ns
						n-3 bal:VPA *vs* n-3 supp:VPA	0.0179	*
						n-3 supp:VPA *vs* n-3 supp:VPA	0.0013	**
	n-3 suppl:SAL	**2-way ANOVA**	F (1, 81)	p value	summary			
		Interaction	1.348	0.249	ns			
		Nutrition	0.6817	0.4114	ns			
		Treatment	1.16	0.2847	ns			
	n-3 bal:VPA	**2-way ANOVA**	F (1, 81)	p value	summary	**Tukey multiple comparisons**	p value	summary
		Interaction	1.161	0.2844	ns	n-3 bal:SAL *vs* n-3 bal:VPA	0.9894	ns
		Nutrition	6.553	0.0123	*	n-3 bal:SAL *vs* n-3 supp:SAL	0.6104	ns
		Treatment	2.482	0.119	ns	n-3 bal:SAL *vs* n-3 supp:VPA	0.0052	**
						n-3 bal:VPA *vs* n-3 supp:SAL	0.9264	ns
						n-3 bal:VPA *vs* n-3 supp:VPA	0.1113	ns
						n-3 supp:VPA *vs* n-3 supp:VPA	0.1319	ns
	n-3 supp:VPA	**2-way ANOVA**	F (1, 81)	p value	summary	**Tukey multiple comparisons**	p value	summary
		Interaction	2.304	0.133	ns	n-3 bal:SAL *vs* n-3 bal:VPA	0.8675	ns
		Nutrition	14.52	0.0003	***	n-3 bal:SAL *vs* n-3 supp:SAL	0.2357	ns
		Treatment	0.3464	0.5578	ns	n-3 bal:SAL *vs* n-3 supp:VPA	0.0355	*
						n-3 bal:VPA *vs* n-3 supp:SAL	0.0443	*
						n-3 bal:VPA *vs* n-3 supp:VPA	0.0068	**
						n-3 supp:VPA *vs* n-3 supp:VPA	0.5529	ns

Supplementary Table S15: Detailed statistics on gut microbiota taxa levels (Bacteroidetes and Firmicutes) for male and female offspring and dams.

	**2-way ANOVA**	F (1, 14)	p value	summary			
**Bacteroidetes Males**	Interaction	0.06148	0.8078	*ns*			
	Nutrition	2.222	0.1582	*ns*			
	Treatment	0.4966	0.4926	*ns*			
	**2-way ANOVA**	F (1, 16)	p value	summary			
**Bacteroidetes Females**	Interaction	0.01486	0.9045	*ns*			
	Nutrition	3.02	0.1015	*ns*			
	Treatment	0.003908	0.9509	*ns*			
	**2-way ANOVA**	F (1, 16)	p value	summary	**Tukey multiple comparisons**	p value	summary
**Bacteroidetes Dams**	Interaction	9.423	0.0073	**	n-3 bal:SAL *vs* n-3 bal:VPA	0.725	*ns*
	Nutrition	9.737	0.0066	**	n-3 bal:SAL *vs* n-3 supp:SAL	>0.9999	*ns*
	Treatment	2.524	0.1317	*ns*	n-3 bal:SAL *vs* n-3 supp:VPA	0.0199	*
					n-3 bal:VPA *vs* n-3 supp:SAL	0.7044	*ns*
					n-3 bal:VPA *vs* n-3 supp:VPA	0.0024	**
					n-3 supp:VPA *vs* n-3 supp:VPA	0.0214	*
	**2-way ANOVA**	F (1, 15)	p value	summary			
**Firmicutes Males**	Interaction	0.2668	0.613	*ns*			
	Nutrition	0.1033	0.7524	*ns*			
	Treatment	0.4678	0.5044	*ns*			
	**2-way ANOVA**	F (1, 15)	p value	summary			
**Firmicutes Females**	Interaction	0.1496	0.7044	*ns*			
	Nutrition	2.596	0.128	*ns*			
	Treatment	2.711	0.1204	*ns*			
	**2-way ANOVA**	F (1, 16)	p value	summary			
**Firmicutes Dams**	Interaction	0.2375	0.6326	*ns*			
	Nutrition	0.008746	0.9267	*ns*			
	Treatment	2.852	0.1107	*ns*			

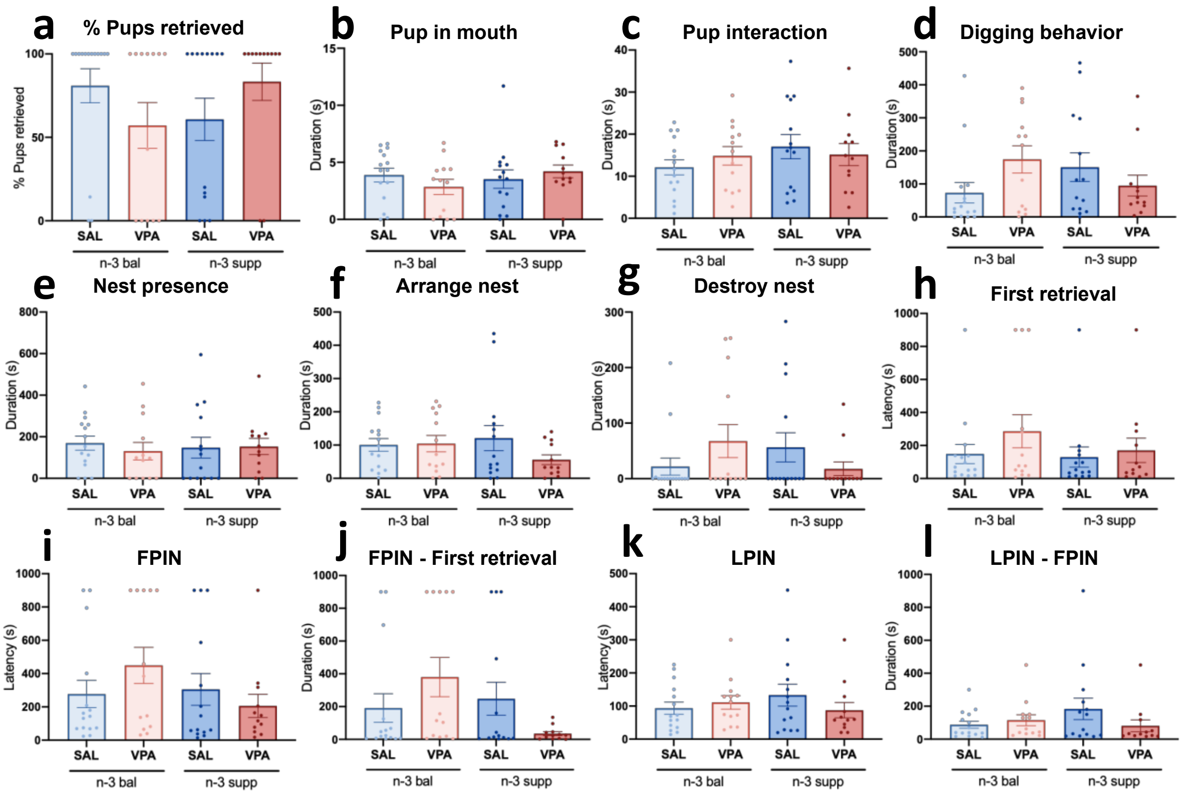

Supplementary Figure S1: Maternal behavior is not affected by VPA treatment or diet. Several parameters were analyzed during the pup retrieval test at P9: **(a)** percentage of pups retrieved, **(b)** time spent with a pup in mouth, **(c)** time spent interacting with pups (i.e. sniffing, touching), **(d)** time spent digging, **(e)** duration of nest presence, **(f)** time spent arranging nest, **(g)** time spent destroying nest, **(h)** latency to first retrieval (pup in mouth), **(i)** latency to first pup in nest (FPIN), **(j)** time spent for the dam from taking the first pup in the mouth to putting it in the nest, **(k)** latency to last pup in nest (LPIN), **(l)** time spent for the dam to retrieve all pups (LPIN-FPIN). n = 15 (SAL/n-3 bal), 13 (VPA/n-3 bal), 14 (SAL/n-3 supp), 12 (VPA/n-3 supp) mice. Data are expressed as mean ± SEM and were analyzed through a two-way ANOVA followed by Tukey post-hoc multiple analysis. *$p < .05$, **$p < .01$, ***$p < .001$.

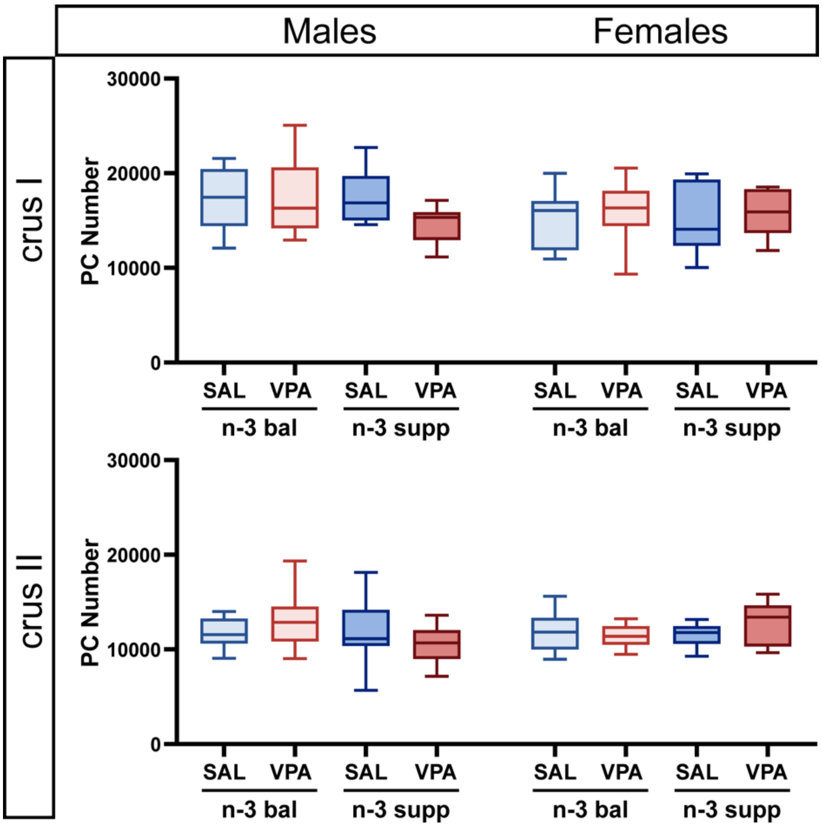

Supplementary Figure S2: VPA does not alter Purkinje cell number in both n-3 balanced and n-3 LCPUFA supplemented diets. PC number in crus I (top) in both males (left) and females (right). n = 11 (SAL/n-3 bal male), 10 (VPA/n-3 bal male), 11 (SAL/n-3 supp male), 9 (VPA/n-3 supp male), 13 (SAL/n-3 bal female), 11 (VPA/n-3 bal female), 7 (SAL/n-3 supp female) and 9 (VPA/n-3 supp female) mice. PC number in crus II (bottom) in both males (left) and females (right). n = 11 (SAL/n-3 bal male), 10 (VPA/n-3 bal male), 12 (SAL/n-3 supp male), 10 (VPA/n-3 supp male), 12 (SAL/n-3 bal female), 11 (VPA/n-3 bal female), 9 (SAL/n-3 supp female) and 9 (VPA/n-3 supp female) mice. Data are expressed as median and min to max and were analyzed through a two-way ANOVA followed by Tukey post-hoc multiple analysis. *$p < .05$, **$p < .01$, ***$p < .001$.

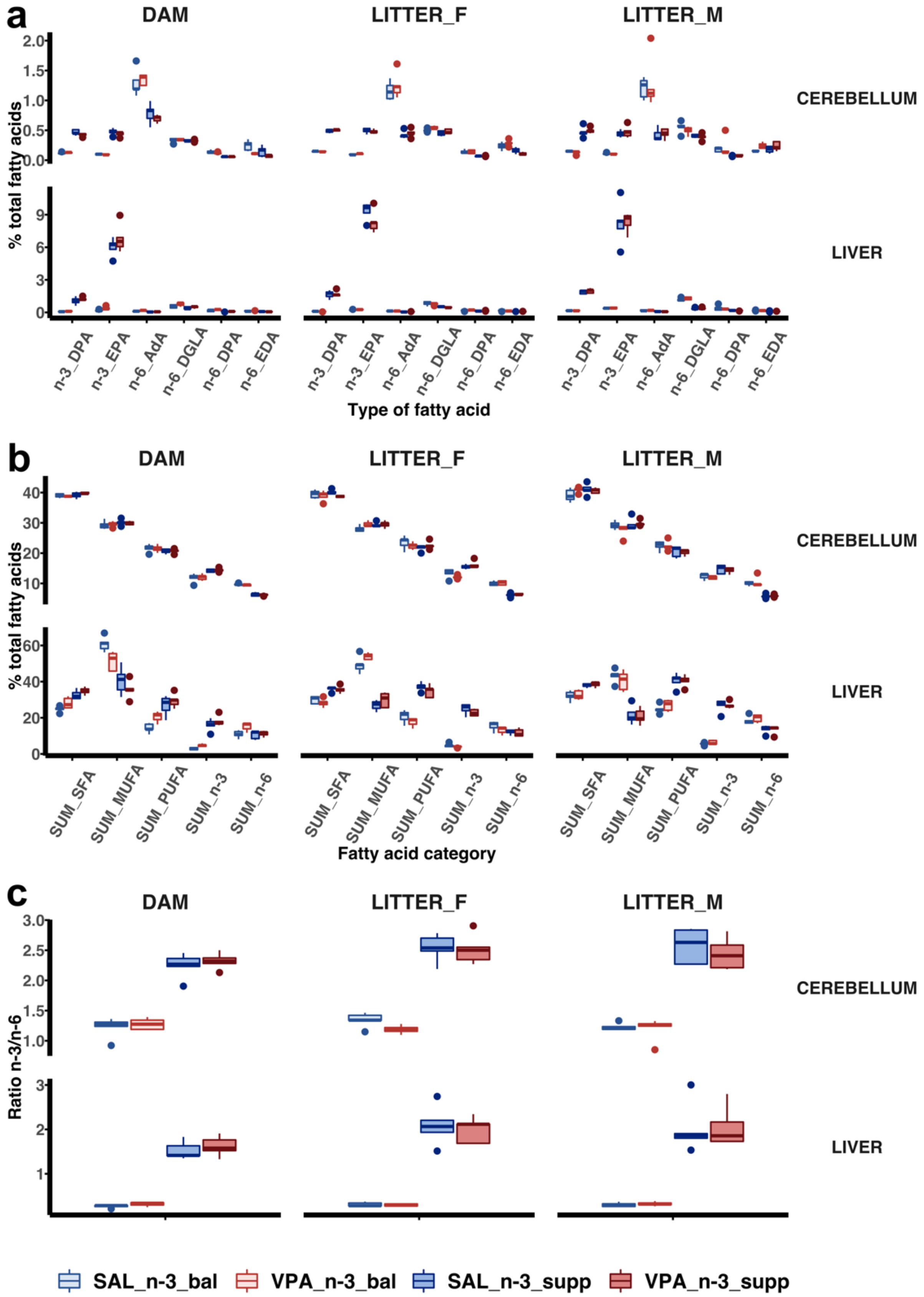

a
b
c
DAM
LITTER_F
LITTER_M
CEREBELLUM
LIVER
% total fatty acids
Type of fatty acid
n-3_DPA
n-3_EPA
n-6_AdA
n-6_DGLA
n-6_DPA
n-6_EDA
Fatty acid category
SUM_SFA
SUM_MUFA
SUM_PUFA
SUM_n-3
SUM_n-6
Ratio n-3/n-6
SAL_n-3_bal
VPA_n-3_bal
SAL_n-3_supp
VPA_n-3_supp

Supplementary Figure S3: Fatty acid composition is different in relation with diet, but not treatment. (a) Different types of fatty acids in the cerebellum and the liver of dams, male and female offspring. **(b)** Different categories of fatty acids in the cerebellum and the liver of dams, male and female offspring. **(c)** n-3/n-6 ratio in the cerebellum and the liver of dams, male and female offspring. n=5 mice per group. DPA: docosapentaenoic acid; EPA: eicosapentaenoic acid; AdA: adrenic acid; DGLA: dihomo-gamma-linolenic acid; EDA: eicosadienoic acid; SFA: saturated fatty acids; MUFA: monounsaturated fatty acids; PUFA: polyunsaturated fatty acids.

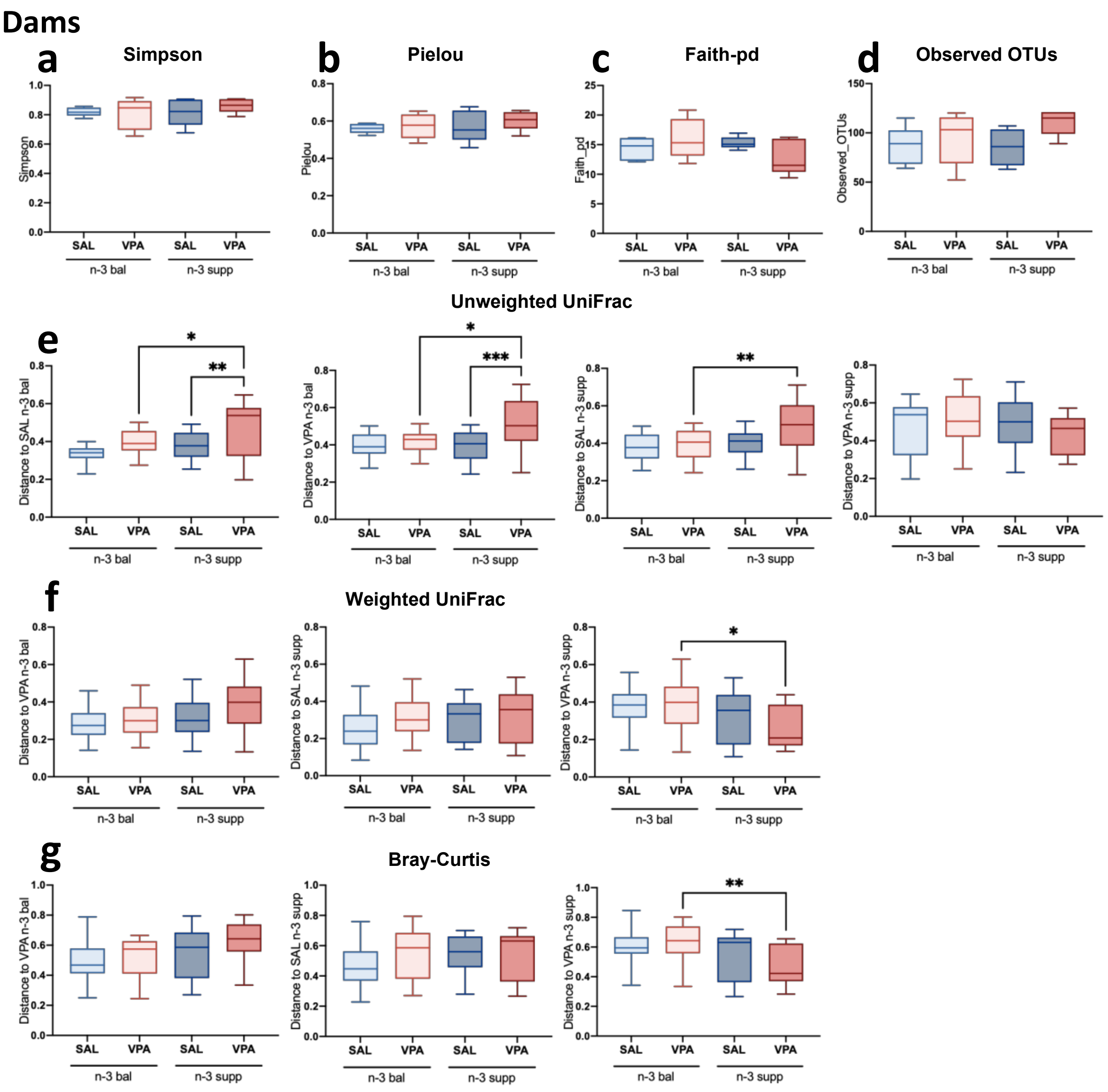

Supplementary Figure S4: Detailed dam microbiota inter- and intra-diversity indexes. (a-c) Alpha diversity indexes: **(a)** Shannon, **(b)** Simpson, **(c)** Pielou and **(d)** Faith-pd **(e-g)** Beta-diversity indexes: **(e)** Unweighted UniFrac, **(f)** Weighted UniFrac and **(g)** Bray-Curtis. n = 5 mice per group. Data are expressed as median and min to max and were analyzed through a two-way ANOVA followed by Tukey post-hoc multiple analysis. *$p < .05$, **$p < .01$, ***$p < .001$.

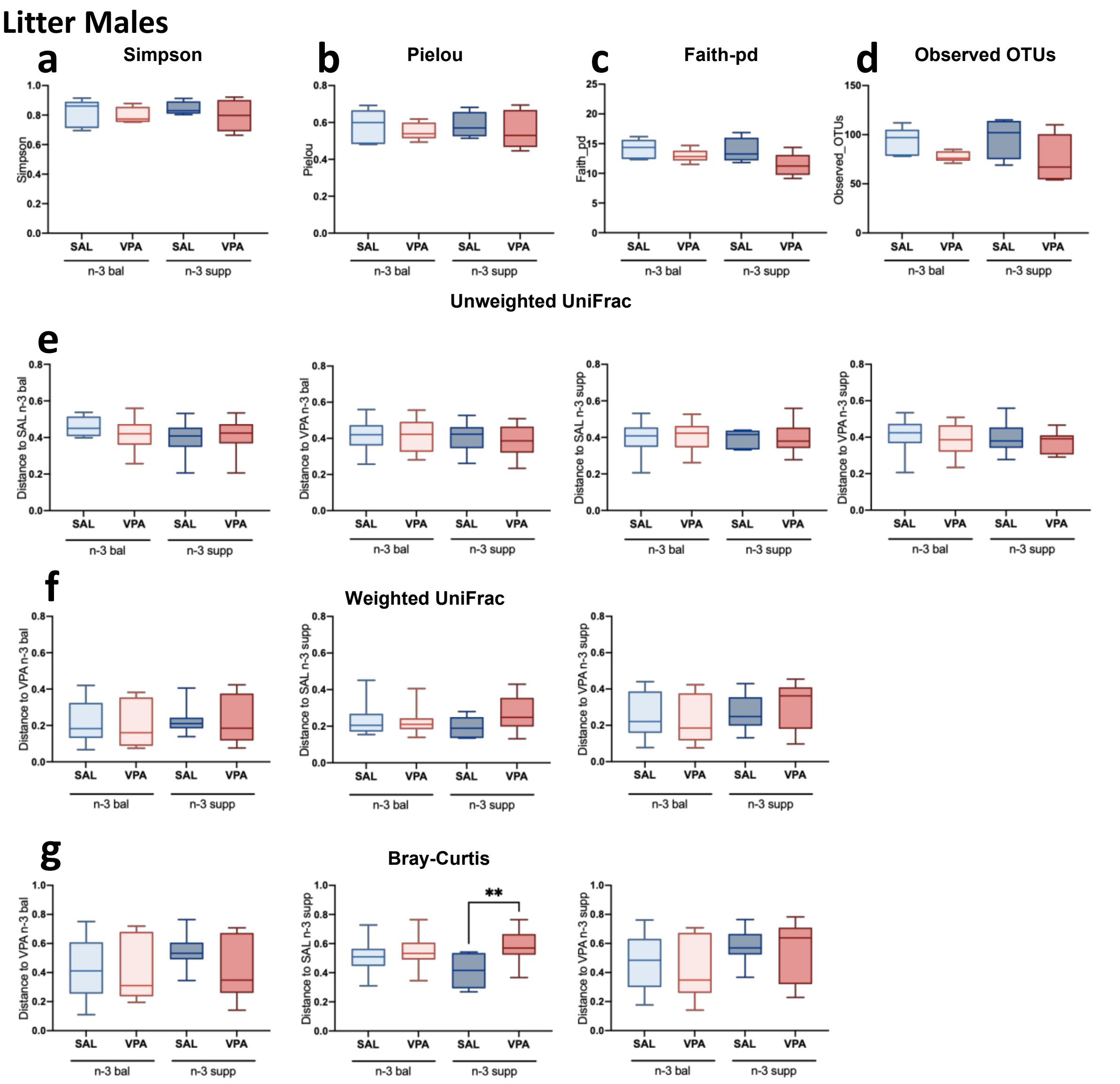

Supplementary Figure S5: Detailed male offspring microbiota inter- and intra-diversity indexes. (a-c) Alpha diversity indexes: **(a)** Shannon, **(b)** Simpson, **(c)** Pielou and **(d)** Faith-pd. **(e-g)** Beta-diverstity indexes: **(e)** Unweighted UniFrac, **(f)** Weighted UniFrac and **(g)** Bray-Curtis. n = 5 mice per group. Data are expressed as median and min to max and were analyzed through a two-way ANOVA followed by Tukey post-hoc multiple analysis. *$p < .05$, **$p < .01$, ***$p < .001$.

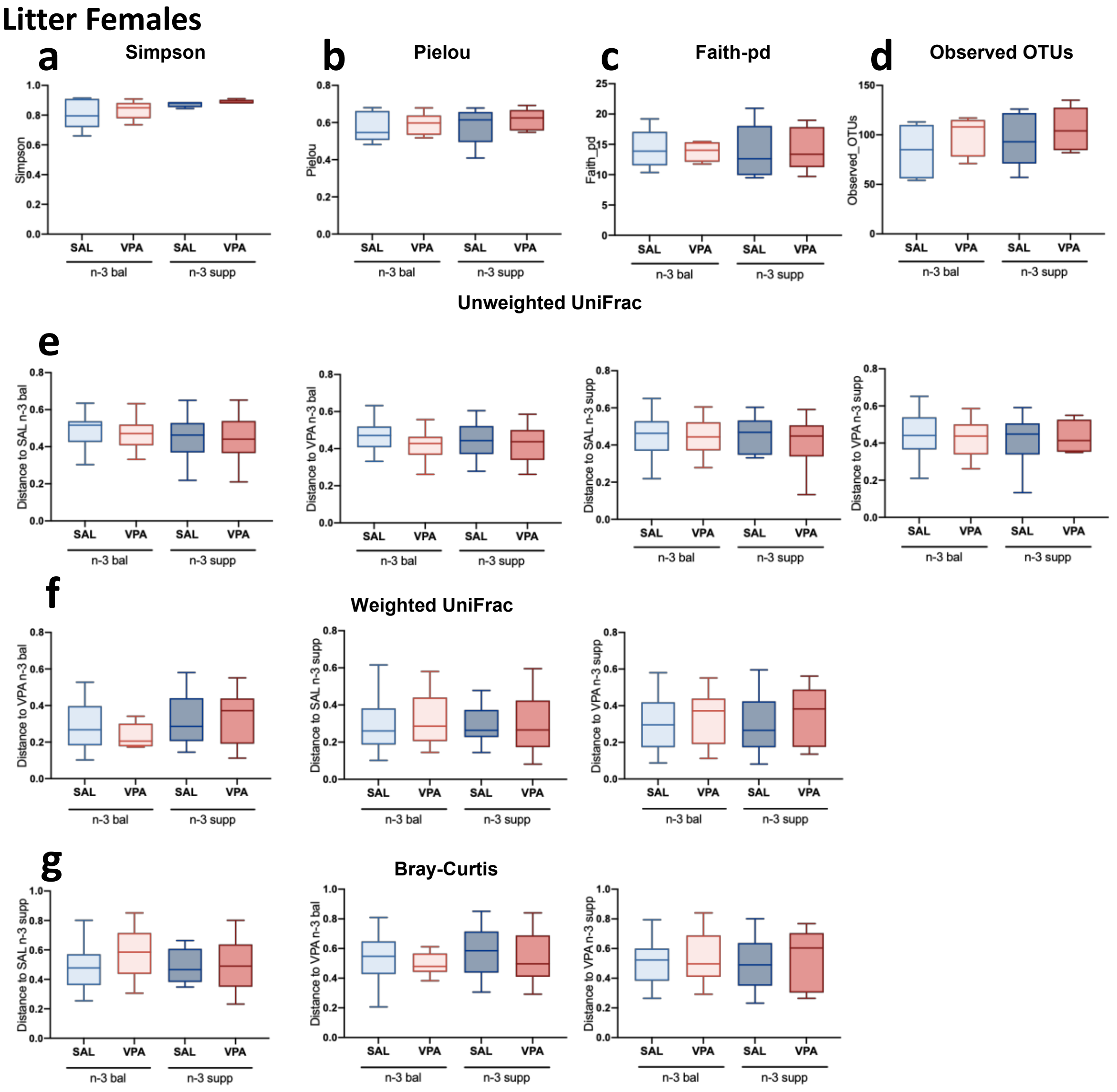

Supplementary Figure S6: Detailed female offspring microbiota inter- and intra-diversity indexes. (a-c) Alpha diversity indexes: **(a)** Shannon, **(b)** Simpson, **(c)** Pielou and **(d)** Faith-pd. **(e-g)** Beta-diverstity indexes: **(e)** Unweighted UniFrac, **(f)** Weighted UniFrac and **(g)** Bray-Curtis. n = 5 mice per group. Data are expressed as median and min to max and were analyzed through a two-way ANOVA followed by Tukey post-hoc multiple analysis. *p < .05, **p < .01, ***p < .001.

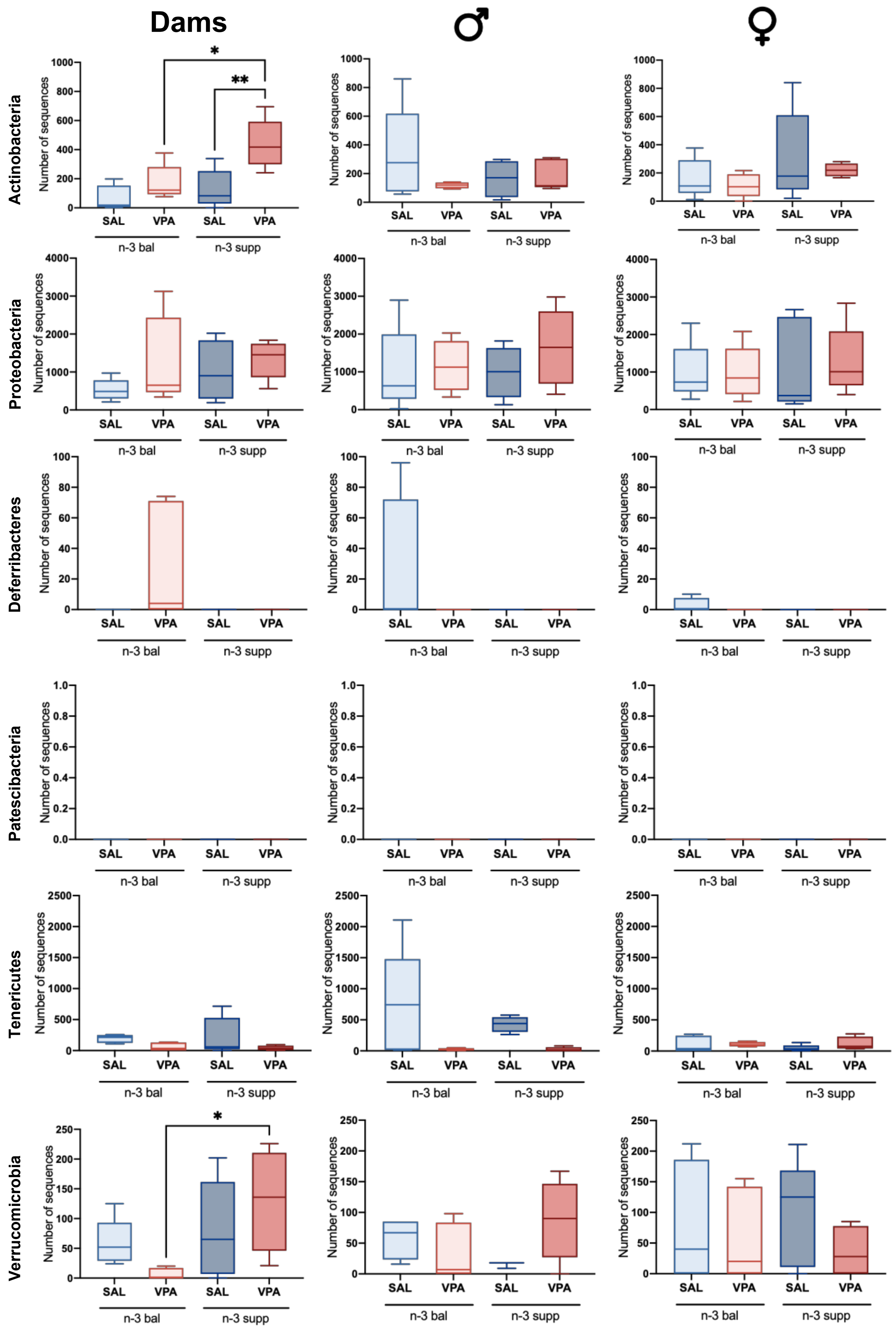

Dams
♂
♀
Actinobacteria
Proteobacteria
Deferribacteres
Patescibacteria
Tenericutes
Verrucomicrobia
Number of sequences
SAL
VPA
n-3 bal
n-3 supp
*
**

Supplementary Figure S7: Dam, male and female offspring microbiota relative abundance. Actinobacteria abundance (first line) in dams (left), offspring males (center) and females (right). n = 5 mice per group except 4 in VPA/n-3 bal and SAL/n-3 supp male and VPA/n-3 supp female. Proteobacteria abundance (second line) in dams (left), offspring males (center) and females (right). n = 5 mice per group except 4 in VPA/n-3 bal and SAL/n-3 supp male. Deferribacteres abundance (third line) in dams (top), offspring males (middle) and females (bottom). n = 5 (SAL/n-3 bal dam), 5 (VPA/n-3 bal dam), 5 (SAL/n-3 supp dam), 5 (VPA/n-3 supp dam), 5 (SAL/n-3 bal male), 5 (VPA/n-3 bal male), 4 (SAL/n-3 supp male), 5 (VPA/n-3 supp male), 4 (SAL/n-3 bal female), 5 (VPA/n-3 bal female), 4 (SAL/n-3 supp female) and 4 (VPA/n-3 supp female) mice. Patescibacteria abundance (fourth line) in dams (top), offspring males (middle) and females (bottom). n = 4 (SAL/n-3 bal dam), 4 (VPA/n-3 bal dam), 5 (SAL/n-3 supp dam), 5 (VPA/n-3 supp dam), 4 (SAL/n-3 bal male), 5 (VPA/n-3 bal male), 4 (SAL/n-3 supp male), 5 (VPA/n-3 supp male), 5 (SAL/n-3 bal female), 4 (VPA/n-3 bal female), 5 (SAL/n-3 supp female) and 4 (VPA/n-3 supp female) mice. Tenericutes abundance (fitth line) in dams (top), offspring males (middle) and females (bottom). n = 5 (SAL/n-3 bal dam), 5 (VPA/n-3 bal dam), 5 (SAL/n-3 supp dam), 4 (VPA/n-3 supp dam), 5 (SAL/n-3 bal male), 4 (VPA/n-3 bal male), 4 (SAL/n-3 supp male), 4 (VPA/n-3 supp male), 5 (SAL/n-3 bal female), 4 (VPA/n-3 bal female), 5 (SAL/n-3 supp female) and 4 (VPA/n-3 supp female) mice. Verrucomicrobia abundance (sixth line) in dams (top), offspring males (middle) and females (bottom). n = 5 (SAL/n-3 bal dam), 5 (VPA/n-3 bal dam), 5 (SAL/n-3 supp dam), 5 (VPA/n-3 supp dam), 5 (SAL/n-3 bal male), 5 (VPA/n-3 bal male), 3 (SAL/n-3 supp male), 5 (VPA/n-3 supp male), 5 (SAL/n-3 bal female), 5 (VPA/n-3 bal female), 5 (SAL/n-3 supp female) and 4 (VPA/n-3 supp female) mice. Data are expressed as median and min to max and were analyzed through a two-way ANOVA followed by Tukey post-hoc multiple analysis. $^{*}p < .05$, $^{**}p < .01$, $^{***}p < .001$.

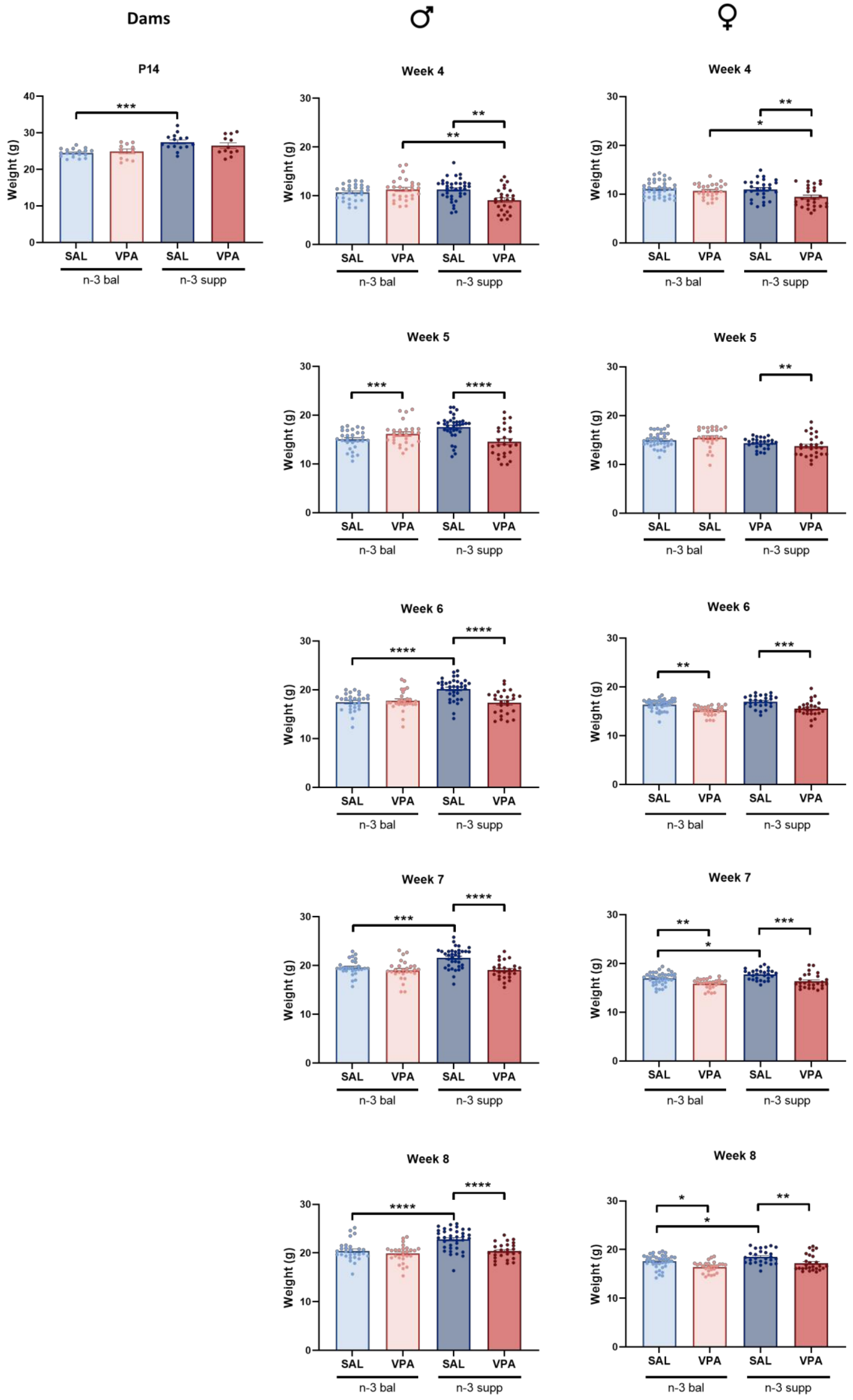

Dams
♂
♀
P14
***
Weight (g)
0
10
20
30
40
SAL
VPA
SAL
VPA
n-3 bal
n-3 supp
Week 4
**
**
Weight (g)
0
10
20
30
SAL
VPA
SAL
VPA
n-3 bal
n-3 supp
Week 4
**
*
Weight (g)
0
10
20
30
SAL
VPA
SAL
VPA
n-3 bal
n-3 supp
Week 5
***
****
Weight (g)
0
10
20
30
SAL
VPA
SAL
VPA
n-3 bal
n-3 supp
Week 5
**
Weight (g)
0
10
20
30
SAL
SAL
VPA
VPA
n-3 bal
n-3 supp
Week 6
****
****
Weight (g)
0
10
20
30
SAL
VPA
SAL
VPA
n-3 bal
n-3 supp
Week 6
**
***
Weight (g)
0
10
20
30
SAL
VPA
SAL
VPA
n-3 bal
n-3 supp
Week 7
***
****
Weight (g)
0
10
20
30
SAL
VPA
SAL
VPA
n-3 bal
n-3 supp
Week 7
**
*
***
Weight (g)
0
10
20
30
SAL
VPA
SAL
VPA
n-3 bal
n-3 supp
Week 8
****
****
Weight (g)
0
10
20
30
SAL
VPA
SAL
VPA
n-3 bal
n-3 supp
Week 8
*
*
**
Weight (g)
0
10
20
30
SAL
VPA
SAL
VPA
n-3 bal
n-3 supp

Supplementary Figure S8: Dam, male and female offspring body weight. Dam body weight (left) at postnatal day 14 (P14; age of the offspring). n = 18 (SAL/n-3 bal dam), 15 (VPA/n-3 bal dam), 11 (SAL/n-3 supp dam) and 12 (VPA/n-3 supp dam). Male offspring body weight (middle) from weaning (Week 4) to the end of the experiment (Week 8). n = 28 (SAL/n-3 bal male), 36 (VPA/n-3 bal male), 27 (SAL/n-3 supp male) and 26 (VPA/n-3 supp male). Female offspring body weight (right) from weaning (Week 4) to the end of the experiment (Week 8). 38 (SAL/n-3 bal female), 27 (VPA/n-3 bal female), 27 (SAL/n-3 supp female) and 26 (VPA/n-3 supp female) mice. Data are expressed as mean ± SEM and were analyzed through a two-way ANOVA followed by Tukey post-hoc multiple analysis. *$p < .05$, **$p < .01$, ***$p < .001$.

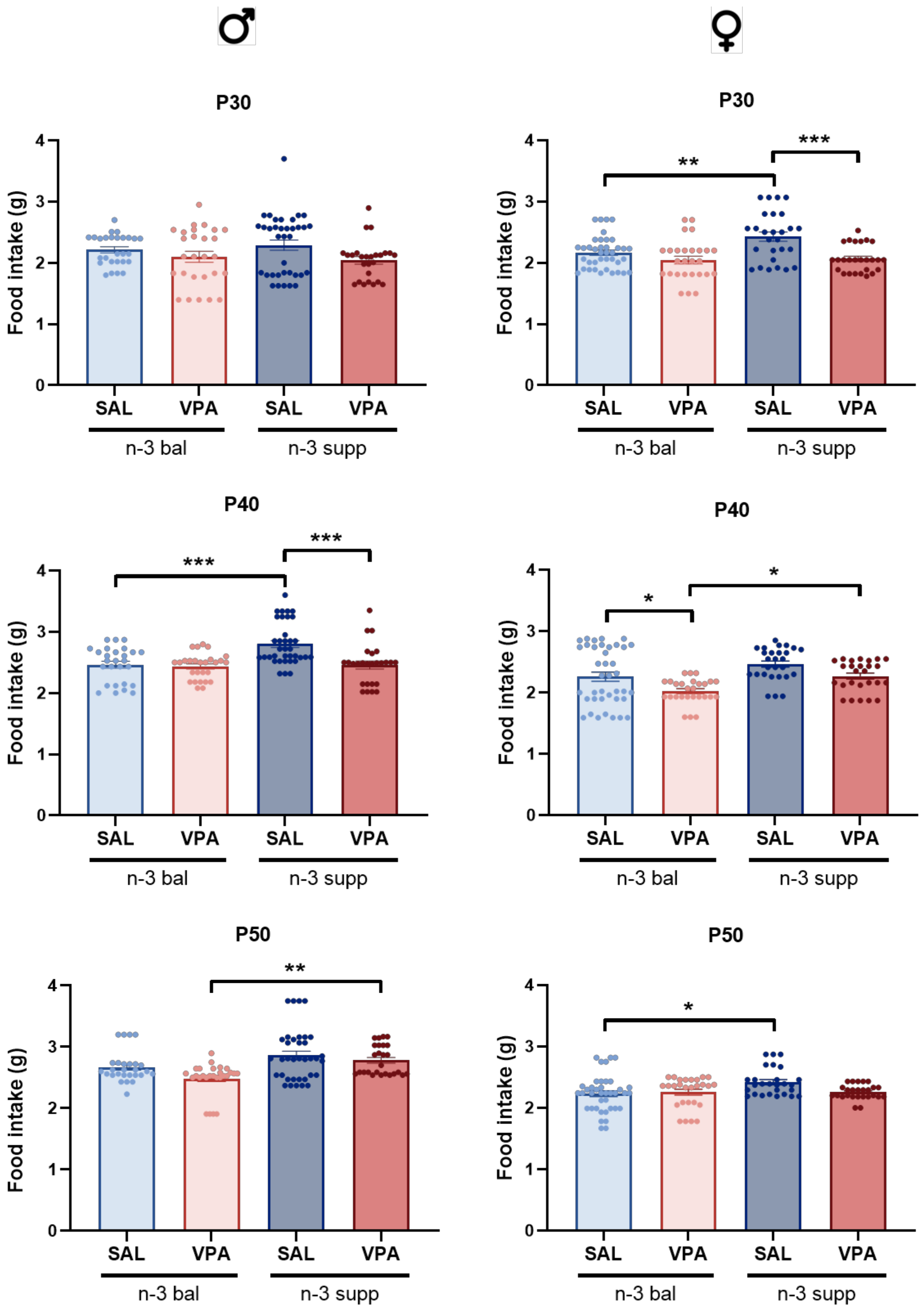

♂
♀
P30
Food intake (g)
0
1
2
3
4
SAL
VPA
SAL
VPA
n-3 bal
n-3 supp
**
***
P40
***
***
*
*
P50
**
*

Supplementary Figure S9: Male and female offspring food intake from weaning to the end of the experiment. Male food intake (left) and female offspring food intake (right) at postnatal days 30, 40 and 50 (P30, P40 and P50). n = 27 (SAL/n-3 bal male), 36 (VPA/n-3 bal male), 36 (SAL/n-3 supp male), 26 (VPA/n-3 supp male), 39 (SAL/n-3 bal female), 27 (VPA/n-3 bal female), 27 (SAL/n-3 supp female) and 26 (VPA/n-3 supp female) mice. Data are expressed as mean ± SEM and were analyzed through a two-way ANOVA followed by Tukey post-hoc multiple analysis. *$p < .05$, **$p < .01$, ***$p < .001$.

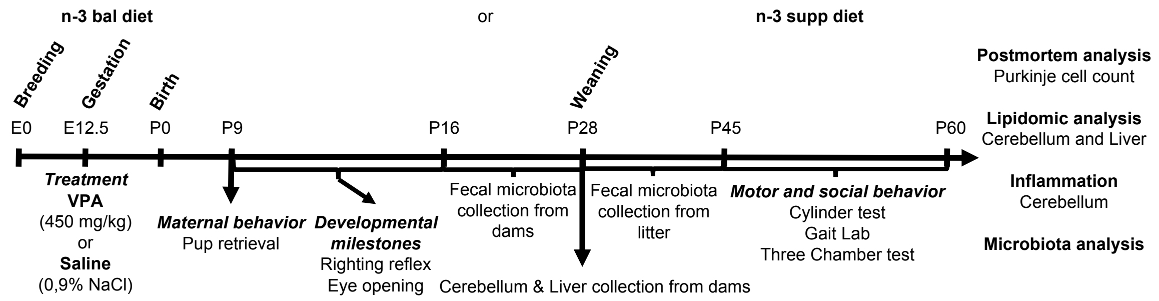

Supplementary Figure S10: Experimental timeline and procedures. C57BL/6J pregnant female mice were fed with a diet enriched with n-3 LCPUFA or with a diet deficient in n-3 LCPUFA from the first gestational day (E0) until weaning of their litter. They received a single intraperitoneal injection of either VPA (450mg/kg) or NaCl 0.9% at gestational day E12.5. Fecal samples were collected both from dams at P15, P20 and P22 and offspring after weaning at P36, P41 and P43. Behavioral investigations on offspring were performed between P9 and P16 (juveniles) and then from P45 to P60 (young adults). This was followed by post-mortem analysis of PC number within the crus I and II cerebellar regions and cerebellar inflammation markers, and for both dams and offspring fatty acid profile in liver and cerebellum and microbiota composition were investigated.
